# Overground robotic training effects on walking and secondary health conditions in individuals with spinal cord injury: systematic review

**DOI:** 10.1186/s12984-022-01003-9

**Published:** 2022-03-15

**Authors:** Federica Tamburella, Matteo Lorusso, Marco Tramontano, Silvia Fadlun, Marcella Masciullo, Giorgio Scivoletto

**Affiliations:** grid.417778.a 0000 0001 0692 3437 I.R.C.C.S. Santa Lucia Foundation (FSL), Via Ardeatina, 306, 00179 Rome, Italy

**Keywords:** Spinal cord injury, Exoskeleton, Robotic, Rehabilitation, Outcome measures

## Abstract

Overground powered lower limb exoskeletons (EXOs) have proven to be valid devices in gait rehabilitation in individuals with spinal cord injury (SCI). Although several articles have reported the effects of EXOs in these individuals, the few reviews available focused on specific domains, mainly walking. The aim of this systematic review is to provide a general overview of the effects of commercial EXOs (i.e. not EXOs used in military and industry applications) for medical purposes in individuals with SCI. This systematic review was conducted following the PRISMA guidelines and it referred to MED-LINE, EMBASE, SCOPUS, Web of Science and Cochrane library databases. The studies included were Randomized Clinical Trials (RCTs) and non-RCT based on EXOs intervention on individuals with SCI. Out of 1296 studies screened, 41 met inclusion criteria. Among all the EXO studies, the Ekso device was the most discussed, followed by ReWalk, Indego, HAL and Rex devices. Since 14 different domains were considered, the outcome measures were heterogeneous. The most investigated domain was walking, followed by cardiorespiratory/metabolic responses, spasticity, balance, quality of life, human–robot interaction, robot data, bowel functionality, strength, daily living activity, neurophysiology, sensory function, bladder functionality and body composition/bone density domains. There were no reports of negative effects due to EXOs trainings and most of the significant positive effects were noted in the walking domain for Ekso, ReWalk, HAL and Indego devices. Ekso studies reported significant effects due to training in almost all domains, while this was not the case with the Rex device. Not a single study carried out on sensory functions or bladder functionality reached significance for any EXO. It is not possible to draw general conclusions about the effects of EXOs usage due to the lack of high-quality studies as addressed by the Downs and Black tool, the heterogeneity of the outcome measures, of the protocols and of the SCI epidemiological/neurological features. However, the strengths and weaknesses of EXOs are starting to be defined, even considering the different types of adverse events that EXO training brought about. EXO training showed to bring significant improvements over time, but whether its effectiveness is greater or less than conventional therapy or other treatments is still mostly unknown. High-quality RCTs are necessary to better define the pros and cons of the EXOs available today. Studies of this kind could help clinicians to better choose the appropriate training for individuals with SCI.

**Supplementary Information:**

The online version contains supplementary material available at 10.1186/s12984-022-01003-9.

## Introduction

The incidence of Spinal Cord Injury (SCI) today is estimated to be from 40 to 80 new cases per million per year worldwide. This means that every year, between 250 000 and 500 000 people become spinal cord injured [1]. SCI is an event that, depending on the level and severity of the injury, has an impact on sensorimotor and autonomous functions.

The main goal of the rehabilitative interventions for individuals with SCI is regaining independence and thus a good quality of life (QoL) [2]. From the patient’s point of view, the level of the SCI could influence regaining a valuable QoL. In fact, considering the shared priorities for individuals with cervical, thoracic or lumbar lesions, the most important factors affecting QoL for individuals with paraplegia are sexual, bowel and bladder functionalities, and for individuals with cervical lesion arm/hand function recovery followed by sexual, bowel and bladder functionalities [3]. Regaining ambulation is also of high priority for individuals with SCI, regardless of the severity, the time after injury and the age at the time of injury [4]. Overall, individuals with SCI have a lower QoL than the general population also because of the presence of Secondary Health Conditions (SHCs). These are referred to physical or psychological conditions directly or indirectly influenced by the presence of a disability or underlying physical impairment [5]. Therefore SHCs due to SCI, such as pain [6], spasticity [4, 6], decreased range of motion (ROM), bowel, bladder [7] and sexual impairments [8], need to be treated in the rehabilitation process [9].

Over the last years, overground powered lower limb exoskeletons (EXOs), most of which were developed exclusively for individuals with SCI [10], have emerged as practical devices for rehabilitative or substitutional interventions. In a rehabilitative framework, EXOs can be used to make multiple steps thus being task-specific for the recovery of walking function. Moreover, individuals using EXOs require good trunk control as well as strength in the upper limbs, in order to balance themselves with the specific devices, and to manage EXOs safely. In cases where rehabilitation of the ambulation is not the aim, the inclusion of the EXO training in the rehabilitation program could serve to train functions such as maintaining a standing position while using upper limbs, practice standing up and sitting down, stimulating trunk movements and other functional tasks that are critical components for achieving functional independence [10]. Previous studies investigating the benefits of both upright posture and mobility show that EXO usage could be beneficial also for bowel functionality [11], chronic pain, spasticity, cardiorespiratory parameters and bone health [12].

Currently, there are few certified over-ground lower limb EXOs for medical use [13]. The EXOs that were approved for use in the US by the Food and Drug Administration are the ReWalk™ [14] (ReWalk Robotics, Inc., Marlborough, MA, USA), the Ekso™ [15] (Ekso Bionics, Richmond, CA, USA), and the Indego™ [16] (Parker Hannifin Corp., Cleveland, OH, USA) devices. The use of ReWalk™ and Indego™ systems was also approved in the European Union (EU). The use of systems by ReWalk™ and Indego™ has been approved in the community and institutional field, while the use of the Ekso™ device has been approved in the medical field, as long as there is a trained medical supervisor. Furthermore, the other EXOs for medical use approved for CE marketing in the EU are Hybrid Assistive Limb—HAL™ [17] (Cyberdine, Tsukuba, Japan) and Rex™ [18] (Rex Bionics Ltd., Aukland, New Zealand). All these EXOs are class II medical devices, each one having specific inclusion and exclusion criteria as well as having been tested in different settings [13].

The EXOs usage and the benefits brought about by it are mostly supported by single-intervention trials with few participants or single case reports. Therefore, it is not possible, to date, to have a clear scientific evidence about the full range of the possible pros and cons, considering also detriments and adverse effects, due to EXOs usage [10]. Furthermore, it has not yet been established if there are domains whit no benefits brought by the EXO training compared to conventional therapy. To date, the available reviews that aimed at shedding light on this topic are few or do not include all commercial EXOs [19, 20]. In addition, these reviews are mainly focused on the mechanical design, the actuation system and the integrated control strategies [21–23] of the EXOs, and on specific issues, such as the effects when using EXOs on walking and endurance [24–27]. Only two reviews, focusing mainly on Ekso, ReWalk and Indego EXOs, addressed the beneficial trends of using EXOs on spasticity, pain or bowel movements regularity [11, 28].

To date there are no systematic reviews collecting the available data on purported functional and health benefits or detriments deriving from the use of EXOs. These not only include walking, but also the SHCs and the impact on the Activities of Daily Living (ADL) or in the QoL. The aim of this systematic review is to provide a clear picture of the existing literature on EXOs’ by exploring the current state of the art of the overground lower limb EXOs and its effects on walking and on SHCs in individuals with SCI. It was conducted in light of the high level of interest for these emerging technological devices, as well as the potential impact on rehabilitation practices and outcomes. EXOs used in the military and industry fields were not targeted in this review, nor were the studies that aimed at addressing the effects of other robotic-assisted gait training for individuals with SCI. To date, the review [29], meta-analysis [30] and clinical practice guideline [31] studies on the effects of robotic-assisted gait training, different from overground EXOs (e.g. body weight supported EXO on treadmill, end effector devices, etc.…) in the framework of SCI rehabilitation, are available. These devices allow individuals to train ambulation in a fixed and confined area with bodyweight support components to facilitate standing [32]. On the contrary, overground EXOs allow individuals to walk exploring the environment, although they require higher balance control and upper limb aids to maintain balance or to control steps initiation [33]. One single review [34], that focused on the effects due to different robotic-assisted gait trainings, highlighted that comparison across devices is difficult due to lack of overground EXO RCTs and to differences across the studies (e.g. neurological and epidemiological features, training protocols and outcome measures).

## Methods

This systematic review was performed in accordance with the PRISMA (Preferred Reporting Items for Systematic Reviews and Meta-Analyses) statement [35].

### Search strategy and eligibility criteria

The following databases were scanned starting without time limits until December 24^th^, 2020. Studies were selected by searching on MED-LINE, Embase, Scopus, Web of Science and Cochrane Library (Cochrane Central Register of Controlled Trials). Keyword terms (“spinal cord diseas*”, “spinal cord injur*”, “robotics”, “exoskeleton”), were combined by using Boolean Operators to search each database. Medical Subject Headings terms (“Spinal Cord Diseases”, “Spinal cord injury”, “Robotics”, “Exoskeleton Device”) were used to search PubMed and the Cochrane Library. English language and human studies were used as restrictions. In addition, hand searches of reference lists from retrieved articles as well as from previously published reviews or meta-analysis, were completed.

Full reports of randomized clinical trials (RCTs) of parallel-group or cross-over design and non-randomized clinical trials (n-RCTs) such as cohort studies, case–control, case series and pilot studies based on Ekso, ReWalk, Indego, Rex and HAL were included. In case of EXO hybrid application (e.g. functional electrical stimulation, transcranial magnetic stimulation, transcranial direct current stimulation, etc., …) corresponding records were excluded. Records were included if at least one session with EXO was performed. In case of EXO training records were included regardless of comparison with conventional physical therapy (CPT) or not. Records based on individuals with SCI over 18 years old, regardless of traumatic or non-traumatic lesion, time since injury (TSI), lesion level, Asia Impairment Scale (AIS) score and sex were selected. Trials that involved people affected by SCI and other neurological conditions (e.g. stroke, multiple sclerosis) were included if at least 50% of participants were affected by a SCI.

### Study selection and data collection process

Duplicate records were identified and removed using the EndNOTE software. Study eligibility assessment and the data extraction process were carried out by two independent co-authors (SF and ML). In case of any disagreement, the opinion of a third author (FT) was used to reach accordance. The first selection of studies was initially conducted based on the title and abstract and afterwards full-text articles were examined.

The summary of results was reported following the PRISMA statement [35]. Two authors (ML and FT) independently extracted the following relevant features of the included studies using a predefined data extraction form: authors; title; year of publication; individuals features (number of participants, sex, age, lesion level, AIS score, TSI, number and reasons of drop-out); exclusion criteria; intervention (EXO, session/treatment duration, frequency, comparison with other rehabilitative approaches); Evaluations: timeline, outcome measures, presence/absence of follow-up; summary of results.

Research design, level of evidence and methodological quality were determined for each included study. Study design was determined according to the Level of Evidence for therapeutic studies following Burns et al. [36]. Scores are detailed in Table 1. Methodological quality score was calculated according to the recognized Downs and Black (D&B) tool [37] which is organized in different subsections: Reporting, External Validity, Internal Validity (bias) and Internal Validity (confounding). Total score ranges from 0 to 28, with a higher score indicating higher methodological quality [38]. According to Singh et al. [39] D&B scores below 11 points indicates “poor” quality; 11–19 points reflects “moderate” quality and > 19 points is considered “good” quality. All included studies were assessed per the D&B tool for methodological quality by two independent raters (FT and ML) that reviewed each article and determined the quality score. Scoring discrepancies were resolved through discussion.

**Table 1:** Five levels of evidence for therapeutic studies (from the Centre for Evidence-Based Medicine, http://www.cebm.net)

Level type of evidence
1A: Systematic review (with homogeneity) of RCTs
1B: Individual RCT (with narrow confidence intervals)
1C: All or none study
2A: Systematic review (with homogeneity) of cohort studies
2B: Individual Cohort study (including low quality RCT, e.g., < 80% follow-up)
2C: “Outcomes” research; Ecological studies
3A: Systematic review (with homogeneity) of case–control studies
3B: Individual Case–control study
4: Case series and poor-quality cohort and case–control study
5: Expert opinion without explicit critical appraisal or based on physiology bench research or “first principles”

## Results

### Identification of studies

**Fig. 1:**
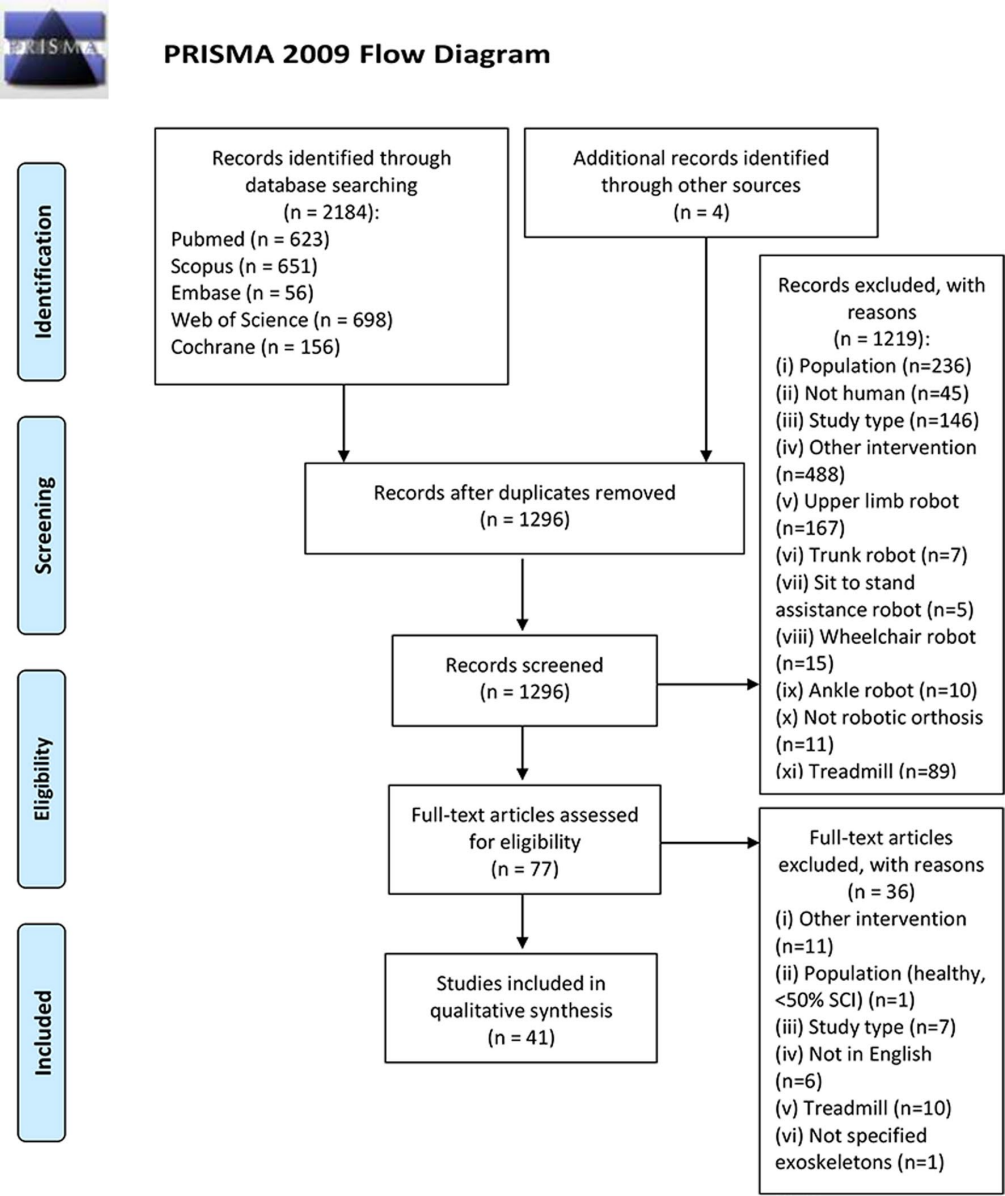
PRISMA flow diagram of the study selection process

A total of 2184 articles were identified from all the considered databases: PubMed (n = 623), Scopus (n = 651), Embase (n = 56), Web of Science (n = 698), Cochrane Library (n = 156), and also 4 articles from other sources were included as additional records. Among these, 888 publications were excluded because they were duplicates. Titles and abstracts were screened for the remaining 1296 articles, 1219 records were excluded because they didn’t satisfy the inclusion criteria (details about reasons for exclusion are reported in Fig. 1). A total of 77 articles were identified as potentially relevant studies, 36 articles were excluded after the full-text review and 41 articles were included. See Fig. 1 for PRISMA flow diagram of the study selection process.

### Levels of evidence and methodological quality

The levels of evidence and the D&B scores for the evaluation of methodological quality are reported in alphabetical order in Table 2. The study design was different across studies and the articles classified with the following evidence levels (see Table 1): 2B (n = 28), 3B (n = 7) and 4 (n = 6). As for methodological quality, the only RCT study [40] included was scored as “moderate” (18/28). For n-RCTs (n = 40) the average total score was 10.32 (± 2.73) out of 28, reflecting “poor” or “moderate” quality. The subsection analysis indicates that the lowest scores were found in Internal validity: the lack of randomization and blinding procedures were the most common issues that caused selection bias. It is worth noting that none of the studies was able to detect significant effects.

**Table 2:** Evidence Level and Downs and Black Tool sub-sections and total scores reported for each study

	Study	Title	Evidence Level	Downs and Black Tool					
				Subsections					Total Score
				Reporting	External Validity	Internal Validity: Bias	Internal Validity: Confounding	Power	
1	Chang et al. 2018	Exoskeleton-assisted gait training to improve gait in individuals with spinal cord injury: A pilot randomized study	2B	7	2	5	4	0	18 (Moderate)
2	Tsai et al. 2020	Exoskeletal-Assisted Walking During Acute Inpatient Rehabilitation Leads to Motor and Functional Improvement in Persons With Spinal Cord Injury: A Pilot Study	3B	10	0	3	2	0	15 (Moderate)
3	Asselin et al. 2015	Heart Rate and Oxygen demand of Powered Exoskeleton-Assisted Walking in person with paraplegia	2B	8	3	3	0	0	14 (Moderate)
4	Gagnon et al. 2018 (A)	Locomotor training using an overground robotic exoskeleton in long-term manual wheelchair users with a chronic spinal cord injury living in the community: Lessons learned from a feasibility study in terms of recruitment, attendance, learnability, performance and safety	2B	8	3	3	0	0	14 (Moderate)
5	Khan et al. 2019	Retraining walking over ground in a powered exoskeleton after spinal cord injury: a prospective cohort study to examine functional gains and neuroplasticity	2B	9	1	3	1	0	14 (Moderate)
6	Platz et al. 2016	Device-Training for Individuals with Thoracic and Lumbar Spinal Cord Injury Using a Powered Exoskeleton for Technically Assisted Mobility: Achievements and User Satisfaction	2B	8	3	3	0	0	14 (Moderate)
7	Baunsgaard et al. 2018 (A)	Gait training after spinal cord injury: safety, feasibility and gait function following 8 weeks of training with the exoskeletons from Ekso Bionics	2B	8	1	3	1	0	13 (Moderate)
8	Baunsgaard et al. 2018 (B)	Exoskeleton gait training after spinal cord injury: An exploratory study on secondary health conditions	2B	8	1	3	1	0	13 (Moderate)
9	van Dijsseldonk et al. 2019	Predictors of exoskeleton motor learning in spinal cord injured patients	2B	7	2	3	1	0	13 (Moderate)
10	Chun et al. 2020	Changes in Bowel Function Following Exoskeletal-Assisted Walking in Persons with Spinal Cord Injury: An Observational Pilot Study	2B	7	3	2	0	0	12 (Moderate)
11	Tefertiller et al. 2018	Initial Outcomes from a Multicenter Study Utilizing the Indego Powered Exoskeleton in Spinal Cord Injury	2B	8	1	3	0	0	12 (Moderate)
12	Yang et al. 2015	Assessment of In-Hospital Walking Velocity and Level of Assistance in a Powered Exoskeleton in Persons with Spinal Cord Injury	2B	8	1	3	0	0	12 (Moderate)
13	Yatsugi et al. 2018	Feasibility of Neurorehabilitation Using a Hybrid Assistive Limb for Patients Who Underwent Spine Surgery	2B	7	2	3	0	0	12 (Moderate)
14	Benson et al. 2016	Lower-limb exoskeletons for individuals with chronic spinal cord injury: Findings from a feasibility study	2B	6	3	2	0	0	11 (Moderate)
15	Escalona et al. 2018	Cardiorespiratory demand and rate of perceived exertion during overground walking with a robotic exoskeleton in long-term manual wheelchair users with chronic spinal cord injury: A cross-sectional study	2B	7	1	3	0	0	11 (Moderate)
16	Fineberg et al. 2013	Vertical ground reaction force-based analysis of powered exoskeleton-assisted walking in persons with motor-complete paraplegia	2B	7	1	3	0	0	11 (Moderate)
17	Guanziroli et al. 2019	Assistive powered exoskeleton for complete spinal cord injury: correlations between walking ability and exoskeleton control	3B	7	1	3	0	0	11 (Moderate)
18	Kubota et al. 2019	Hybrid assistive limb (HAL) treatment for patients with severe thoracic myelopathy due to ossification of the posterior longitudinal ligament (OPLL) in the postoperative acute/subacute phase: A clinical trial	2B	8	0	3	0		11 (Moderate)
19	Sale et al. 2016 (A)	Effects on mobility training and de-adaptations in subjects with Spinal Cord Injury due to a Wearable Robot: A preliminary report	4	8	0	3	0	0	11 (Moderate)
20	Stampacchia et al. 2016	Walking with a powered robotic exoskeleton: Subjective experience, spasticity and pain in spinal cord injured persons	2B	7	1	3	0	0	11 (Moderate)
21	Zeilig et al. 2012	Safety and tolerance of the ReWalkTM exoskeleton suit for ambulation by people with complete spinal cord injury: A pilot study	4	7	1	3	0	0	11 (Moderate)
22	Alamro et al. 2018	Overground walking with a robotic exoskeleton elicits trunk muscle activity in people with high-thoracic motor-complete spinal cord injury	3B	7	0	3	0	0	10 (Poor)
23	Esquenazi et al. 2012	The ReWalk Powered Exoskeleton to Restore Ambulatory Function to Individuals with Thoracic-Level Motor-Complete Spinal Cord Injury	2B	7	0	2	1	0	10 (Poor)
24	Juszczak et al. 2018	Examining the Effects of a Powered Exoskeleton on Quality of Life and Secondary Impairments in People Living With Spinal Cord Injury	2B	6	1	3	0	0	10 (Poor)
25	Karelis et al. 2017	Effect on body composition and bone mineral density of walking with a robotic exoskeleton in adults with chronic spinal cord injury	2B	7	0	3	0	0	10 (Poor)
26	Kozlowski et al. 2015	Time and effort required by persons with spinal cord injury to learn to use a powered exoskeleton for assisted walking	2B	7	1	2	0	0	10 (Poor)
27	McIntosh et al. 2020	The Safety and Feasibility of Exoskeletal-Assisted Walking in Acute Rehabilitation After Spinal Cord Injury	4	6	1	3	0	0	10 (Poor)
28	Ramanujam et al. 2018 (A)	Neuromechanical adaptations during a robotic powered exoskeleton assisted walking session	3B	7	0	3	0	0	10 (Poor)
29	Sale et al. 2018 (B)	Training for mobility with exoskeleton robot in spinal cord injury patients: a pilot study	2B	7	0	3	0	0	10 (Poor)
30	Birch et al. 2017	Results of the first interim analysis of the RAPPER II trial in patients with spinal cord injury: ambulation and functional exercise programs in the REX powered walking aid	2B	7	1	1	0	0	9 (Poor)
31	Evans et al. 2015	Acute Cardiorespiratory and Metabolic Responses During Exoskeleton-Assisted Walking Overground Among Persons with Chronic Spinal Cord Injury	2B	6	0	3	0	0	9 (Poor)
32	Gagnon et al. 2019 (B)	Satisfaction and perceptions of long-term manual wheelchair users with a spinal cord injury upon completion of a locomotor training program with an overground robotic exoskeleton	2B	6	0	3	0	0	9 (Poor)
33	Hartigan et al. 2015	Mobility outcomes following five training sessions with a powered exoskeleton	2B	6	1	2	0	0	9 (Poor)
34	Lonini et al. 2016	Accelerometry-enabled measurement of walking performance with a robotic exoskeleton: a pilot study	3B	5	1	3	0	0	9 (Poor)
35	Ramanujam et al. 2018 (B)	Mechanisms for improving walking speed after longitudinal powered robotic exoskeleton training for individuals with spinal cord injury	3B	6	0	3	0	0	9 (Poor)
36	Kressler et al. 2014 (A)	Understanding therapeutic benefits of overground bionic ambulation: exploratory case series in persons with chronic, complete spinal cord injury	4	6	0	2	0	0	8 (Poor)
37	Kolakowsky-Hayner et al. 2013	Safety and Feasibility of using the EksoTM Bionic Exoskeleton to Aid Ambulation after Spinal Cord Injury	2B	5	1	1	0		7 (Poor)
38	Kressler et al. 2019 (B)	Cardiometabolic Challenges Provided by Variable Assisted Exoskeletal Versus Overground Walking in Chronic Motor-incomplete Paraplegia: A Case Series	4	5	0	2	0	0	7 (Poor)
39	Manns et al. 2019	Perspectives of people with spinal cord injury learning to walk using a powered exoskeleton	2B	5	0	1	0	0	6 (Poor)
40	Talaty et al. 2013	Differentiating ability in users of the ReWalk(TM) powered exoskeleton: an analysis of walking kinematics	3B	3	0	0	0	0	3 (Poor)
41	Cahill et al. 2018	Gym-based exoskeleton walking: A preliminary exploration of non-ambulatory end-user perspectives	4	2	0	0	0	0	2 (Poor)

### Participants

**Fig. 2:**
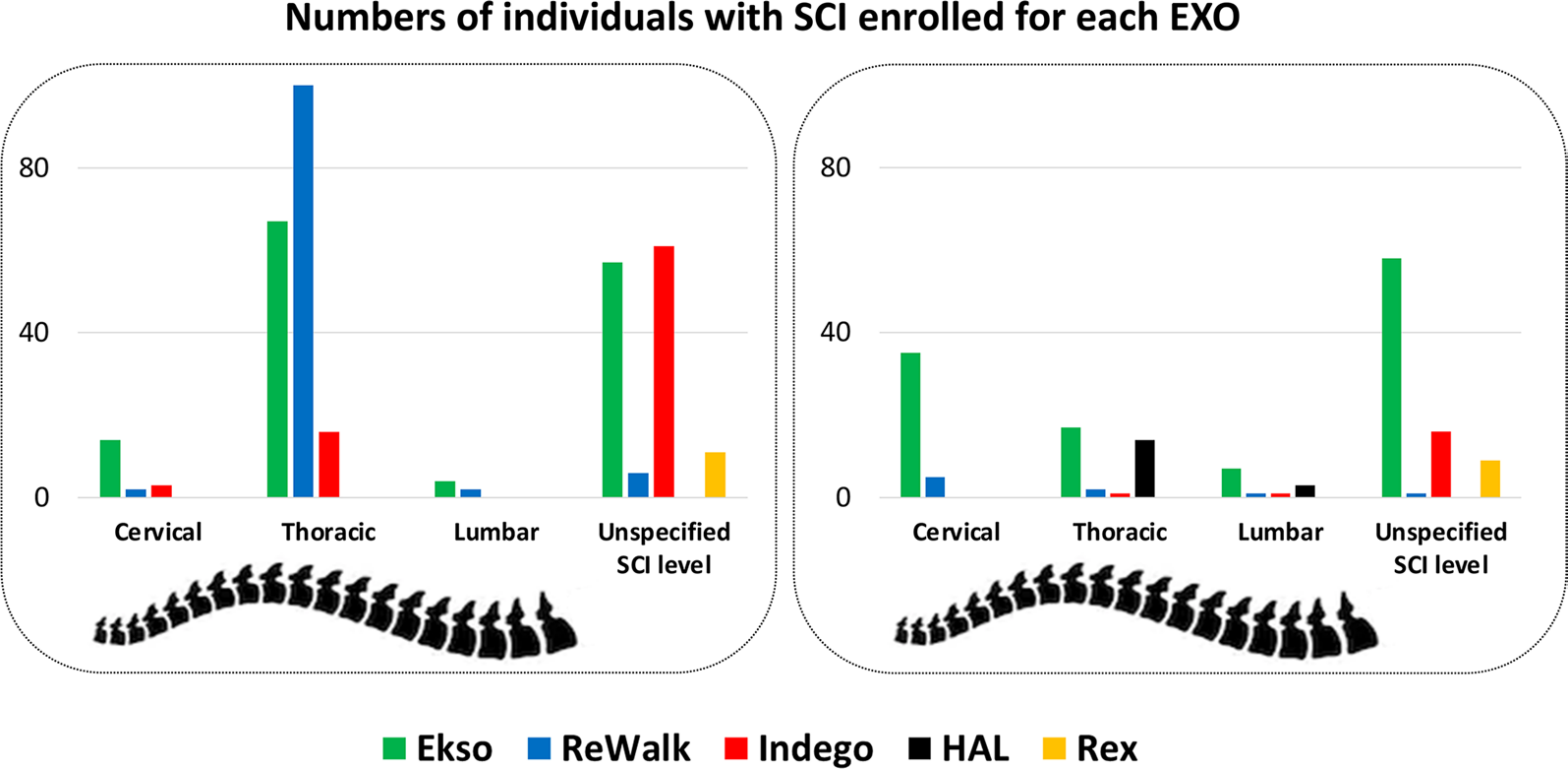
Number of individuals with SCI enrolled according to the lesion level (cervical, thoracic or lumbar SCI) across the 41 included studies. Ekso device: green columns; ReWalk device: blue columns; Indego device: red columns; HAL device: black columns; Rex device: orange columns. Individuals are grouped into AIS A plus AIS B group (left frame) and AIS C plus AIS D group (right frame)

A total of 580 participants were included. Two studies [41, 42] discussed the effects on the same population, therefore, after removing duplicates, a total sample of 566 participants (M = 411, F = 143 and one study didn’t describe participants’ sex [43]) was analyzed. The average age of participants was 43.58 years ± 7.84. Specifically, the recruited population included 25 able-bodied subjects (ABs), 348 motor complete injuries (AIS A and B) and 170 motor incomplete injuries (AIS C and D). Two studies [43, 44] did not specify AIS level. Details about number of individuals with SCI with cervical, thoracic or lumbar lesions grouped according to AIS level are reported in Fig. 2. Participants were in subacute (i.e. less than six months after SCI) (N = 115) or in chronic stages (N = 325), and 4 studies [45–48] did not specify the TSI (N = 101). None of the 41 included studies, enrolled individuals with diseases other than SCI. Participants’ data is reported in Table 3. Exclusion criteria of individuals with SCI are reported in “Additional file 1”.

**Table 3:** Participants and intervention data classified according to EXOs and time since injury

		Study (D&B total score)	Number of participants (Sex)	Individuals features	Exoskeleton Intervention	Comparison Intervention	Evaluations	Follow-up	Drop out
*Exoskeleton: Ekso*									
Subacute	n-RCT	Tsai et al. 2020 (15)	30 (24 M, 6F)	LOI: C-T-L AIS A(3), B(3), C(13), D(11) Age: mean 46.8 years (intervention group), mean 52 years (control group) TSI: mean 21.3 days (intervention group), mean 17.4 days (control group)	Part of minimum 15 h of CPT x week	CPT	Each session: up time, walk time, steps (reported as mean across all training sessions) Pre and Post training: UEMS, LEMS, FIM	NP	NR
		McIntosh et al. 2020 (10)	11 (8 M, 3F)	LOI: C6-L2 AIS A(5), C(5), D(1) Age: mean 41 years TSI: 3–15 weeks	1 h × 3 days/week × 25 sessions (in associaton with CPT)	NO	Each session: up time, walk time, steps number; Pre and Post each session: VAS_p; Sitting vs standing vs prior to sitting: BP, HR; Sitting, session mid-time, prior to resitting: BRPE (1–10); Sessions 2, 13, 25: 6MWT, 10MWT	NP	1
Chronic	RCT	Chang et al. 2018 (18)	7 (5 M, 2F)	LOI: C4-T12 AIS C(2), D(5) Age: mean 56 years intervention group), mean 60 years (control group) TSI: 15 years (intervention group), 7 years (control group)	1 h × 5 days/week × 3 weeks	CPT	Pre and Post training: stride length, step length, cadence, 6MWT, 10MWT, LEMS, TUG	NP	2
	n-RCT	Gagnon et al. 2018 (A) (14)	14 (9 M, 5F)	LOI: C6-T10 AIS A(14) Age: mean 38.7 years TSI: mean 7.4 years	1 h × 3 days/week × 18 sessions	NO	Each session: up time, walk time, steps number, level of assistance provided by trained assistant during walking Pre and Post training: 10MWT	NP	1
		Escalona et al. 2018 (11)	13 (8 M, 5F)	LOI: C6-T10 AIS A(13) Age: mean 38.1 years TSI: mean 5.1 years	2–3 days/week × 18 sessions	NO	Single evaluation at last session while sitting, standing, walking: VO2, VCO2, VE, VT, RER, RR, HR Single evaluation at last session after walking: BRPE (1–10)	NP	0
		Sale et al. 2016 (A) (11)	3 (2 M, 1F)	LOI: T6-L1 AIS A(2), C(1) Age: mean 36 years TSI: Chronics	50 min × 3–4 days/week × 20 sessions	NO	Pre and Post training: velocity, cadence, step width, step length, stance time, double support time, Satisfaction Questionnaire, 6MWT indoor, 6MWT outdoor, 10MWT, TUG, VAS_p, VAS fatigue, BRPE(1–10)	NP	0
		Alamro et al. 2018 (10)	16 (8 ABs, 8 SCI) (11 M, 5F)	LOI: C7-T4 AIS A(6), B(2) Age: mean 38.7 years TSI: 1–25 years	1 session: 3 walking conditions	Ekso-OG, Ekso on treadmill and Lokomat	Single evaluation: trunk muscle activation, trunk acceleration	NP	0
		Karelis et al. 2017 (10)	5 (4 M, 1F)	LOI: C7-T10 AIS A(5) Age: mean 60.4 years TSI: mean 7.6 years	1 h × 3 days/week × 6 weeks	NO	Pre and Post training: Body Weight, BMI, Total lean body mass, Arm lean body mass, Leg lean body mass, Appendicular lean body mass, Trunk lean body mass, Total fat mass, Arm fat mass, Leg fat mass, Appendicular fat mass, Trunk fat mass, Total bone mineral density, Leg bone mineral density, Tibia Bone mineral density, Cross-sectional area of the calf Muscle, Subcutaneous adipose tissue, Intramuscular adipose tissue	NP	0
		Ramanujam et al. 2018 (A) (10)	8 (4 Abs, 4 SCI) (6 M, 2F)	LOI: C5-T10 AIS A(2), C(2) Age: mean 41.75 years (individuals with SCI) 27.25 years (ABs) TSI: 0.5–9.5 years	1 session	AB: walking without Ekso at self-selected, fast and slow speeds and with Ekso in "Passive" and "Active" conditions	Single evaluation: walking velocity, stance time, swing time, mean knee and hip ROM, EMG lower limbs	NP	NR
		Gagnon et al. 2018 (B) (9)	14 (9 M, 5F)	LOI: C6-T10 AIS A, B, C Age: mean 38.7 years TSI: mean 7.4 years	Questionnaire after 1 h × 2–3 days/week × 18 sessions	NO	Post training: questionnaire	NP	NR
		Ramanujam et al. 2018 (B) (9)	12 (4 Abs, 8 SCI) (9 M, 3F)	LOI: C4-T11 AIS NR (incomplete) Age: mean 39.12 years TSI: mean 6.38 years	3–4 days/week × 100 h	AB: walking with Ekso	Pre and Post training: stride time, step time, stance time, double support time, stride length, step length, step frequency, walking speed	NP	NR
		Kressler et al. 2014 (A) (8)	3 (2 M, 1F)	LOI: T1-T10 AIS A(3) Age: mean 30.33 years TSI: ≥ 1 yr	1 h × 3 days/week × 6 weeks	NO	Pre, Mid and Post training: 10MWT, 2MWT, SCATS, ISCIBPD, NRS_p, EMG, %VO2 peak, EE	NP	NR
		Kressler et al. 2019 (B) (7)	2 (1 M, 1F)	LOI: T6-T12 AIS NR (incomplete) Age: mean 45 years TSI: 2–9 years	1 session walking with Ekso and 1 session walking without Ekso	Overground walking	Single evaluation for each session: VO2, HR, EE	NP	NR
		Cahill et al. 2018 (2)	4 (3 M, 1F)	LOI: NR AIS NR (2 complete, 2 incomplete) Age: mean 41 years TSI: mean 5 years	13–25 months	NO	Post training: semi-structured interview	NP	NR
Subacute + chronic	n-RCT	Baunsgaard et al. 2018 (A) (13)	52 (36 M, 16F)	LOI: C1-L2 AIS A and B (36), C and D (16) Age: mean 35.8 years TSI: 0.2–10.8 years	3 days/week × 8 weeks	NO	Each session: up time, walk time, steps number, BRPE(6–20) Pre, Mid and Post training: up time, walk time, steps number, 10MWT, TUG, BBS, WISCI II, LEMS, HR and BP (before and after 10 min of walking)	4 weeks: LEMS, 10MWT, TUG, BBS, WISCI II	8
		Baunsgaard et al. 2018 (B) (13)	52 (36 M, 16F)	LOI: C1-L2 AIS A and B (36), C and D (16) Age: mean 35.8 years TSI: 0.2–10.8 years	20–60 min × 3 days/week × 8 weeks	NO	After each session, Pre, Mid and Post training: ISCIPBDS, MAS Pre and Post trainingt: hip, knee and ankle flexor/extensor ROM, SCIM III, ISCIBDS for bowel, bladder and QoL	4 weeks: ROM, SCIM III, ISCIBDS for bowel, bladder and QoL	NR
		Stampacchia et al. 2016 (11)	21 (17 M, 4F)	LOI: C7-L2 AIS A(12), B(2), D(7) Age: mean 48.1 years TSI: 2–330 months	40 min × 1 session	NO	Pre and Post single session: MAS, PSFS, NRS_sp, NRS_p Post session: PGIC, ad hoc questionnaire for subjective experience	NP	NR
		Kozlowski et al. 2015 (10)	7 (7 M)	LOI: C4-L1 AIS A(3), B(1), C(3) Age: mean 36 years TSI: 0.4–7.4 years	2 h × 1–2 days/week x up to 24 sessions	NO	Number of sessions needed to achieve a rating of “minimal assistance” and to achieve “contact guard” for walking and stand/sit Each session (only best performance was reported): walk time, up time, steps number, walk distance during longest walk and 2MWT, donning/doffing assistance Sitting, session mid-time, after resitting: BP, HR, METs, BRPE(6–20)	NP	NR
		Kolakowsky-Hayner et al. 2013 (7)	7 (5 M, 2F)	LOI: T4-T12 AIS A(7) Age: mean 29.8 years TSI: 65–578 days	1 h × 1 day/week × 6 weeks	NO	Each session: up time, walk time, step length, distance, don/doff time, level of assistance provided by trained assistant during walking, SPS, loss of balance	NP	1
Unspecified TSI	n-RCT	Sale et al. 2018 (B) (10)	8 (6 M, 2F)	LOI: T1-L2 AIS A(3), B(4), C(1) Age: mean 43.25 years TSI: NR	45 min × 5–6 days/week × 20 sessions	NO	Pre and Post each session: HR, BP Pre and Post training: 6MWT indoor/outdoor, BRPE, 10MWT, cadence, stride length, walking velocity, stance phase, swing phase, double support, pelvis tilt initial contact, ROM pelvis tilt, hip, knee and ankle flexion/extension ROM, TUG, VAS_p, VAS fatigue, Satisfaction Questionnaire	NP	0
Total of Ekso studies			275 (199 M, 76F)	LOI: Cervical (49), Thoracic (84), Lumbar (11), NR (115) AIS: A + B(164), C + D(95) Age: mean 42.55 years (individuals with SCI), mean 27.5 years (ABs) TSI: subacute (98), chronic (153), NR (8)					
*Exoskeleton: ReWalk*									
Chronic	n-RCT	Asselin et al. 2015 (14)	8 (7 M, 1F)	LOI: T2-T11 AIS A(7) B(1) Age: mean 46.2 years TSI: 5.9 years	60–90 min × 1 session	NO	Single evaluation while sitting, standing and walking: VO2, HR After walking: BRPE(6–20)	NP	NR
		Khan et al. 2019 (14)	12 (8 M, 4F)	LOI: C6-T10 AIS A(6), B(2), C(3), D(1) Age: mean 37.5 years TSI: mean 7.6 years	12 weeks	NO	Each session: total steps number, steps without stopping, walking distance, walking speed Pre and Post each session: NRS_p Pre and Post training: UEMS, LEMS, MEP, sensory key-points ISNCSCI Pre, Mid and Post training: CoP limits of stability and sway speed Weekly: SCATS, McGill Pain Questionnaire Pain Rating Index Post training: 10MWT, 6MWT During 6MWT and wheelchair propulsion: PCI	Between 2 and 3 months: 10MWT, 6MWT, CoP limits of stability and sway speed	3
		Platz et al. 2016 (14)	7 (5 M, 2F)	LOI: T-L AIS A(6), C(1) Age: mean 48.3 years TSI: mean 11.4 years	1 h × 5 days/week × 4–5 weeks	NO	Number of session to achieve with physical help/verbal assistance/no assistance: sit to stand, stand to sit, standing balance 1 min with crutches, walk 10 mt straight, walk 10 m straight and in curve, ascend, turn around, descend 12 stairs, walk 500 m outdoor Pre and Post training: REPAS, LEMS, UEMS, ASIA sensory examination, SCIM, SF-12v2	1 month: SF-12v2	0
		van Dijsseldonk et al. 2019 (13)	20 (12 M, 8F)	LOI: T AIS A(19), B(1) Age: mean 37 years TSI: mean 8 years	1.5 h × 3 days/week × 8 weeks	NO	Pre and Post training and every 2 weeks (2,4,6): evaluation of potential predictors (neurological lesion level, age, gender, age at injury onset, time since injury, physical activity level, level of anxiety and BMI)	NP	4
		Chun et al. 2020 (12)	11 (10 M, 1F)	LOI: T2-T11 AIS A(9), B(2) Age: 18–65 years TSI: 1–15 years	30–90 min × 3–4 days/week × 12–14 weeks	NO	Pre and Post training: Modified Lynch Gastrointestinal Survey, Bristol Stool Scale, SCI-QOL Bowel Management DIfficulties	NP	1
		Yang et al. 2015 (12)	12 (10 M, 2F)	LOI: C8-T11 AIS A(9), B(2), C(1) Age: 16–75 years TSI: 1–20 years	1–2 h x mean 55 sessions	NO	Best performance: correlation between level of assistance provided by trained assistant during walking versus 6MWT and 10MWT	NP	NR
		Benson et al. 2016 (11)	10 (10 M)	LOI: C8-L1 AIS A(7), C(3) Age: mean 31.7 years TSI: 1–21 years	2 h × 2 days/week × 10 weeks	NO	Pre and Post each session: HR, BP, AS, VAS_p, VAS fatigue Pre and Post Training: ISNCSCI, 10MWT, 6MWT, TUG, ADAPSS, ATD-PA	NP	5
		Fineberg et al. 2013 (11)	9 (3 AB, 6 SCI) (7 M, 2F)	LOI: T1-T11 AIS A(5), B(1) Age: mean 44.83 years individuals with SCI), 41.67 years (ABs) TSI: 1.5–14 years	1–2 h × 3 days/week × 5–6 months	AB walking wit ReWalk	Single evaluation after reaching the ability to walk 10 m: vGRF, walking velocity	NP	NR
		Guanziroli et al. 2019 (11)	15 (11 M, 4F)	LOI: T4-L5 AIS A(15) Age: mean 39.3 years TSI: 6 months-15 years	1 h × 3 days/week × 8 weeks (at least)	Comparison between two generations of ReWalk software	Single evaluation after training: 6MWT, 10MWT, STS-time	NP	2
		Zeilig et al. 2012 (11)	6 (6 M)	LOI: T5-T12 AIS A(6) Age: mean 33.16 years TSI: 3–7 years	50 min × 13.7 ± 5.8 sessions	NO	Pre and Post Training: VAS fatigue, VAS_p Each session: HR, BP, VAS fatigue, VAS_p Post Training (comparison between high vs low lesions): 10MWT, 6MWT, TUG, Satisfaction Questionnaire	NP	2
		Esquenazi et al. 2012 (10)	12 (8 M, 4F)	LOI: T3-T12 AIS A(12) Age: mean 38.6 years TSI: 1–24 years	75–90 min × 3 days/week × 8 weeks	NO	Pre and Post each session: HR, BP, AS, VAS_p, VAS fatigue Post Training: Satisfaction Questionnaire, 6MWT, 10MWT	12–15 months (data not analyzed)	0
		Lonini et al. 2016 (9)	11 (6 AB—5 SCI) (6 M, 5F)	LOI: T8-T10 AIS A(5) Age: mean 36.9 years TSI: 10 months-7 years	1 h × 3 days/week × 6–12 weeks	Expert users: 6 ABs and 1 individual with SCI	Each session: steps frequency, steps number, EE, trunk angle Pre and Post training: 10MWT, Hip and Knee Flexion, Swing Time, Step Delay, Walking Speed	NP	0
		Manns et al. 2019 (6)	11 (7 M, 4F)	LOI: NR AIS NR Age: mean 37.5 years TSI: mean 7.8 years	60–90 min × 4 days/week × 12 weeks	NO	Pre and Post training: semi-structured interviews on "contributing, changing and learning"	2 months: semi-structured interview on "contributing, changing and learning"	NR
		Talaty et al. 2013 (3)	12	LOI: C7-T12 AIS NR Age: NR TSI: Chronics	60–90 min × 3 days/week × 24 sessions	NO	Single evaluation near training conclusion: comparison among fast vs medium vs slow velocity of flexion/extension ROM of trunk, hip, knee and ankle	NP	NR
Total of ReWalk studies			156 (107 M, 37F, 12 NR)	LOI: Cervical (7), Thoracic (107), Lumbar (3), NR (30) AIS: A + B(115), C + D(9), NR(23) Age: mean 39.18 years (individuals with SCI), mean 39.79 years (ABs) TSI: chronic (147)					
*Exoskeleton: Indego*									
Chronic	n-RCT	Evans et al. 2015 (9)	5 (4 M, 1F)	LOI: T6-T12 AIS A(5) Age: mean 42 years TSI: Chronics	2 sessions	NO	Comparison between 1 session of 6MWT at comfortable speed vs 1 session of 6MWT at "fast but safe" speed: 6MWT, % Vo2 peak, Vo2 average,HR peak, Walking economy, MET	NP	NR
Subacute + chronic	n-RCT	Juszczak et al. 2018 (10)	45 (37 M, 8F)	LOI: T1-L2 AIS A(30), B(5), C(10) Age: mean 35 years TSI: Subacute/Chronics	3–4 days/week × 8 weeks	NO	Before each session: NRS_p, NRS_sp Pre and Mid and Post training: donning/doffing time, NRS spasticity, MAS, indoor/outdoor BRPE (6–20), SWLS, Self reported Bowel and Bladder perception	NP	NR
Unspecified TSI	n-RCT	Tefertiller et al. 2018 (12)	32 (27 M, 5F)	LOI: T4-L2 AIS A(21), B(5), C(6) Age: mean 37 years TSI: NR	3 days/week × 8 weeks	NO	Mid and Post training: indoor/outdoor 10MWT, 6MWT, TUG, Donn/doff time Single evaluation between Mid and Post training: 600MWT	NP	0
		Hartigan et al. 2015 (9)	16 (13 M, 3F)	LOI: C5-L1 AIS A(11), B(3), C(2) Age: 18–51 years TSI: NR	1.5 h × 5 sessions	NO	Last session: 10MWT, 6MWT, level of assistance provided by trained assistant during walking, donn/doff time	NP	NR
Total of Indego studies			98 (81 M, 17F)	LOI: Cervical (3), Thoracic (17), Lumbar (1), NR (77) AIS: A + B(80), C + D(18) Age: mean 38 years TSI: chronic (5), NR (93)					
*Exoskeleton: HAL*									
Subacute	n-RCT	Yatsugi et al. 2018 (12)	9 (6 M, 3F)	LOI: C2-L5 AIS incomplete Age: mean 53.6 years TSI: Subacute	50 min x mean 5 sessions x mean 6 days	NO	Pre and Post training: 10MWT, Cadence, GARS-M, BI, WISCI II, Maximun lateral trunk swing angle during gait	NP	NR
		Kubota et al. 2019 (11)	8 (4 M, 4F)	LOI: T2-T12 AIS D(8) Age: mean 60.9 years TSI: Subacute	1 h × 2–3 days/week × 10 sessions (in association with CPT)	NO	Each session, Pre and Post-training: 10MWT, Cadence, Step lenght, WISCI II, LEMS, FIM	NP	0
Total of HAL studies			17 (10 M, 7F)	LOI: Thoracic (14), Lumbar (3) AIS: C + D(17) Age: mean 57.25 years TSI: subacute (17)					
*Exoskeleton: Rex*									
Chronic	n-RCT	Birch et al. 2017 (9)	20 (14 M, 6F)	LOI: C4-L5 AIS A and B (11), C and D (9) Age: mean 40.9 years TSI: 1–52 years	3–4 h × 1 session	NO	Single evaluation: time to transfer into device, level of assistance provided by trained assistant to perform 2 excersises with upper extremities, TUG, Acceptability questionnaire	NP	NR

Studies data are hierarchically reported according to the Down and Black total (D&B tool) score for each EXO device. NP, not performed follow-up assessment; NR, not reported data

**Fig. 3:**
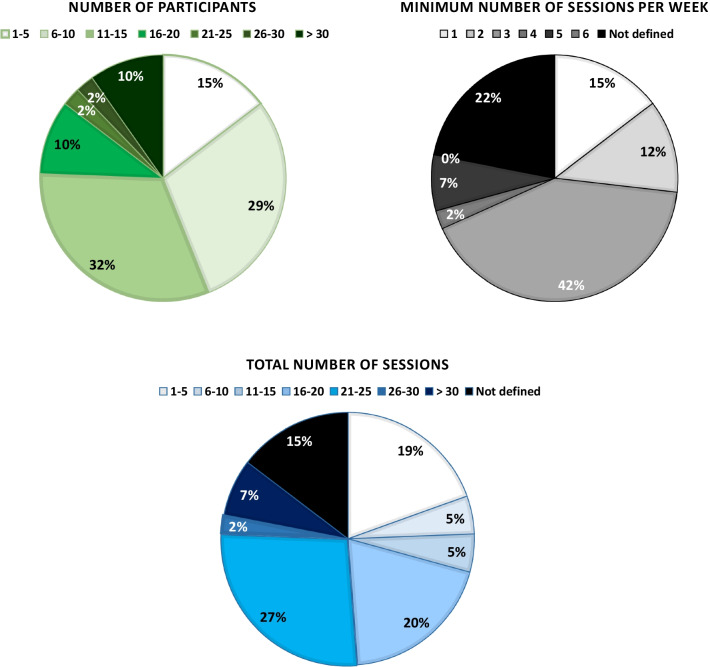
Percentage distributions of number of participants, minimum number of sessions per week and total number of sessions across the 41 included studies

275 participants were enrolled for Ekso studies, 98 in subacute phase, 153 in a chronic stage and 16 were ABs. For the remaining 8 Ekso participants TSI was not defined. Regarding ReWalk, 9 ABs and 147 individuals with chronic SCI were recruited. For Indego 98 individuals were recruited, only for 5 individuals the TSI was indicated (i.e. chronic SCI). The 2 studies conducted using HAL analyzed 17 individuals with subacute SCI, while the single Rex study was conducted on 20 individuals with chronic SCI. The number of participants in the different studies was variable ranging from N = 2 to N = 52. For details, see Fig. 3.

A total of 30 drop-outs was registered, of which 12 were males, 2 were females, and the sex of the remaining was not specified. The reasons for drop-outs were: residence location (N = 4), adverse events with ankle swelling (N = 3), fractures of foot bone (N = 2), recurrent skin breakdowns (N = 2), participants disliked using the device (N = 2), concurrent medical conditions (N = 2), didn’t complete training program (N = 4), other motivations (N = 5), and no reasons specified (N = 3).

For the 41 studies included, 13 studies reported different adverse events during training with Ekso (N = 5), ReWalk (N = 5), Indego (N = 2) and HAL (N = 1) devices. The most frequent adverse events were skin lesions, while the less frequent ones were the presence of extreme fatigue, falls, bone fractures or muscle strain. Details about the adverse events are reported in Table 4. Eleven studies stated that no adverse events occurred during training while the remaining 17 studies did not address the presence or absence of such.

**Table 4:** Adverse events occurred during EXO training

EXO	Study	Skin lesion	Dizziness or Syncope	Swelling or Edema or Bruising	Soreness or Pain	Orthostatic Hypothension	Extreme fatigue or Sprain	Fall	Bone fracture	Muscle strain	Other
Ekso	Tsai et al. 2020	✓									
	Chang et al. 2018				✓						
	Gagnon et al. 2018 (A)/(B)				✓	✓			✓		
	Ramanujam et al. 2018 (B)										
	Baunsgaaard et al. 2018 (A)	✓	✓	✓							
ReWalk	Khan et al. 2019	✓						✓		✓	✓ Trainer Adverse Event
	Platz et al. 2016	✓		✓	✓						
	Yang et al. 2015	✓									
	Benson et al. 2016	✓							✓		
	Esquenazi et al. 2012	✓	✓	✓							
Indego	Tefertiller et al. 2018	✓					✓				
	Hartigan et al. 2015	✓		✓							
HAL	Kubota et al. 2019	✓					✓				

Only studies for which adverse events were reported are listed; “✓” indicates the presence of adverse events

### Intervention

Ekso device effects were analyzed in 20 studies [40–42, 48–64], ReWalk ones in 14 studies [12, 43, 44, 65–75], Indego ones in 4 [45–47, 76], HAL and Rex devices respectively were analyzed in 2 [77, 78] and 1 studies [79]. Ekso studies were conducted on individuals with subacute (N = 2 [49, 53]) and chronic (N = 12 [40–42, 50, 51, 54–60]) lesions. In 5 studies a population with mixed TSI (subacute and chronic SCI) was recruited [52, 61–64]. In the remaining 1 study [48] TSI was not specified. The 14 ReWalk studies involved only individuals with chronic SCI [12, 43, 44, 65–75]. Indego device effects were analyzed in 4 studies [45–47, 76], that enrolled individuals with chronic lesion (N = 1) [76], mixed TSI (N = 1) [46]; while 2 [45, 47] studies did not specify the TSI. The 2 HAL studies focused exclusively on individuals with subacute SCI [77, 78], while the single Rex study assessed only individuals with chronic TSI [79].

EXOs training protocols concerning number of sessions, frequency and duration are reported in Table 3. The average total number of sessions across the studies ranged from 1 to 55. As for session frequency, 42% of the studies included performed 3 sessions per week (see Fig. 3).

### Comparison

Group comparison was present in 6 studies [52, 61, 65, 69, 72] and was extremely heterogeneous (see Table 3). Only for Ekso studies was available the comparison between EXO trainings vs other interventions: Ekso training vs CPT [40] in individuals with chronic SCI and Ekso training plus CPT vs CPT alone [53] in individuals with subacute SCI. Two more studies focusing on Ekso [59] and Indego [76] devices compared performances of individuals with SCI walking in two different conditions: with and without EXO. Performances in walking with ReWalk device of inexperienced individuals with SCI were compared to expert individuals with SCI and vs ABs [70]. Moreover, a comparison among Ekso overground walking vs Ekso treadmill walking vs Lokomat device was available [55]. Regarding follow-up examinations, these assessments were performed 4 weeks after the end of treatment (N = 3) [52, 61, 72] or after 2 months (N = 1) [44], 2–3 months (N = 1) [65] and 12–15 months (N = 1) [69].

### Outcome measures

**Fig. 4:**
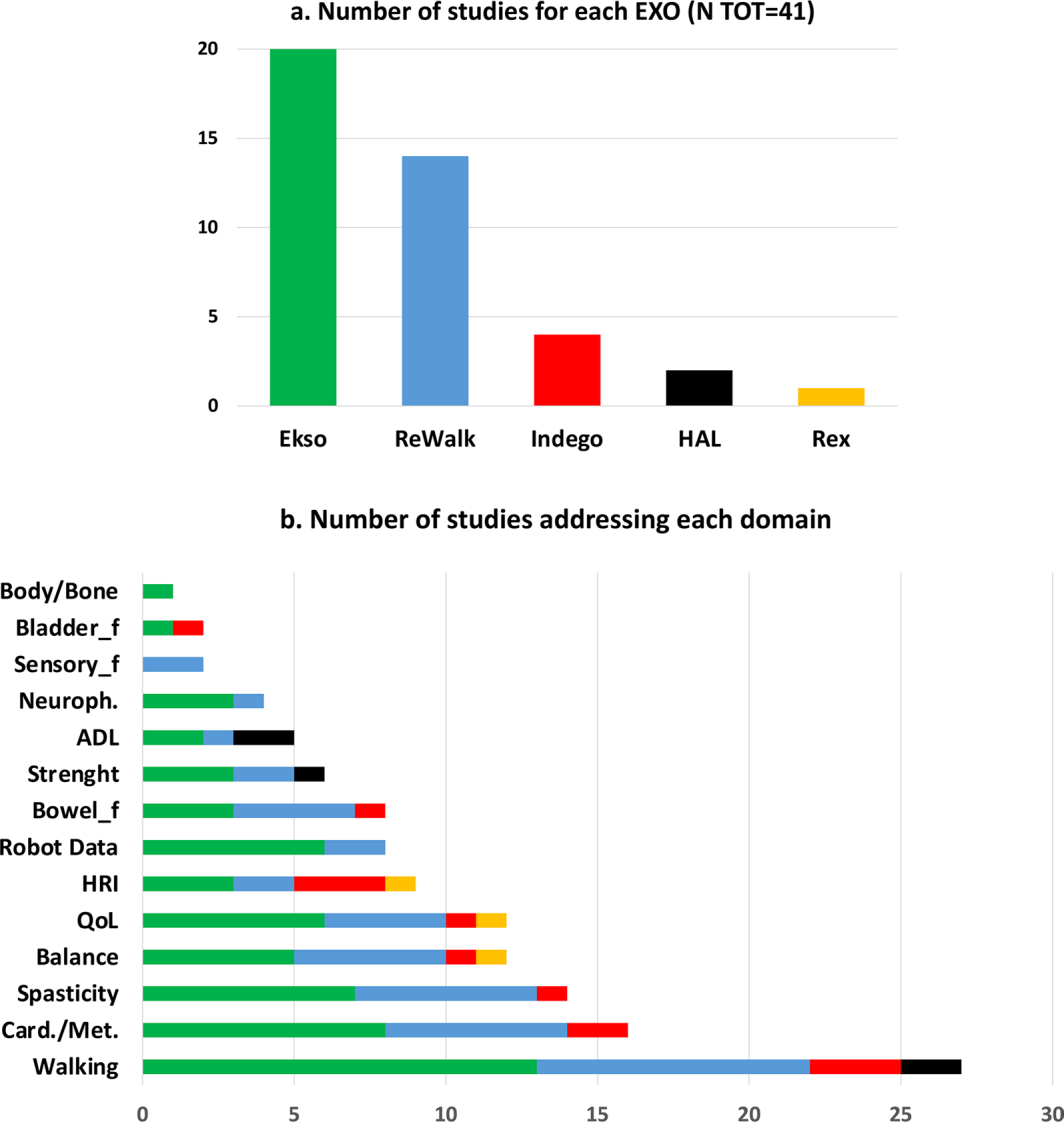
Number of the studies included in the review for each EXO (**a**) and number of studies addressing each domain (**b**). Ekso device: green columns; ReWalk device: blue columns; Indego device: red columns; HAL device: black columns; Rex device: orange columns. [*Card./Met.: Cardiorespiratory and Metabolic responses; QoL: Quality of Life; HRI: Human Robot Interaction; Bowel_f: Bowel functionality; ADL: Activities of Daily Living; Neuroph.: Neurophysiology; Sensory_f: Sensory function; Bladder_f: Bladder functionality; Body/Bone: Body composition and bone density*]

In the included studies, different outcome measures were addressed covering various domains. For comparison purposes, studies were grouped in 14 domains as detailed in Fig. 4 and Tables 5 and 6.

Most of the enrolled studies used outcome measures relating to the walking domain (N tot = 27; N = 13 Ekso, N = 9 ReWalk, N = 3 Indego, N = 2 HAL). Other domains were less addressed. Sixteen studies reported cardiorespiratory and metabolic outcome measures (N = 8 Ekso, N = 6 ReWalk, N = 2 Indego), spasticity and related outcomes were addressed in 14 studies (N = 7 Ekso, N = 6 ReWalk, N = 1 Indego). Balance (N = 5 Ekso, N = 5 ReWalk, N = 1 Indego, N = 1 Rex) and QoL outcome measures were present in 12 studies (N = 6 Ekso, N = 4 ReWalk, N = 1 Indego, N = 1 Rex). Human Robot Interaction (HRI) (N = 9; N = 3 Ekso, N = 2 ReWalk, N = 3 Indego, N = 1 Rex), Robot data (N = 8; N = 6 Ekso, N = 2 ReWalk), and bowel functionality (N = 8; N = 3 Ekso, N = 4 ReWalk, N = 1 Indego) were addressed respectively by 9 and 8 studies. Very little attention was paid to muscle strength (N tot = 6; N = 3 Ekso, N = 2 ReWalk, N = 1 HAL), Activities of Daily Living (N tot = 5; N = 2 Ekso, N = 1 ReWalk, N = 2 HAL) and neurophysiology data (N tot = 4; N = 3 Ekso, N = 1 ReWalk). Finally, almost no attention was given to sensory functions (N = 2 ReWalk) and bladder functionality (N tot = 2, N = 1 Ekso, N = 1 Indego) as well as to body composition and bone density (N = 1 Ekso). It is worth noting that for individuals with no walking function (i.e. non–ambulatory) all evaluations were performed wearing the EXO, while for those who were able to walk overground at evaluation time (i.e. ambulatory), assessments were performed not wearing the EXO (see Tables 5, 6).

**Table 5:** Results for the six most addressed domains classified according to EXO and time since injury

		Study	Walking	Cardiorespiratory and metabolic responses	Spasticity	Balance	Quality of Life	Human Robot Interaction
*Exoskeleton: Ekso*								
Subacute	n-RCT	Tsai et al. 2020						
		McIntosh et al. 2020	*Session 2, 13, 25: 6MWT ↗, 10MWT ↘*	*Longitudinal evaluation:* *BP and HR: Sitting < Standing < After walking; BRPE (1–10) ↗*	Longitudinal evaluation: VAS_p ↘			
Chronic	RCT	Chang et al. 2018	Post vs Pre training: **stride length ↑; step length ↑; 6MWT ↑;** cadence ↑; 10MWT ↓			Post vs Pre training: TUG ↓		
	n-RCT	Gagnon et al. 2018 (A)	*Post vs Pre training: ****10MWT ↓***					Longitudinal evaluation: level of assistance provided by trained assistant during walking ↘
		Escalona et al. 2018		*Single evaluation:* ***HR, VO2, VCO2, RR and VE: Sitting < Standing < Walking;*** ***RER: Sitting > Standing < Walking; VT: Sitting < Walking;*** *Single evaluation after walking: BRPE(1–10) not compared*				
		Sale et al. 2016 (A)	*Post vs Pre training:* ***velocity ↑; cadence ↑; 6MWT indoor/outdoor ↑;**** 10MWT ↓; step length ↑; step width ↑; stance time ↑, double support time (rigth ↑, left ↓)*	*Post vs Pre training:* *BRPE (1–10) ↓; VAS fatigue ↓*	Post vs Pre training: VAS_p ↓	*Post vs Pre training: TUG ↓*	Post vs Pre training: Satisfaction questionnaire ↑	
		Alamro et al. 2018	*Single evaluation: t****runk medial–lateral/anterior–posterior acceleration Ekso-OG > trunk medial–lateral/anterior–posterior acceleration Lokomat;*** *Trunk acceleration: no differences between Ekso-OG vs Ekso on treadmill*					
		Karelis et al. 2017						
		Ramanujam et al. 2018 (A)	*Single evaluation:* ***walking velocity: SCI < ABs passive condition; stance time: SCI > ABs active condition;*** *walking velocity: ABs with Ekso < ABs without Ekso; ROM: SCI < ABs active condition*					
		Gagnon et al. 2019 (B)					Post training: on line questionnaire not compared	
		Ramanujam et al. 2018 (B)	*Post vs Pre training individuals with SCI: walking speed ↑, stride time ↓, stance time ↓, double support ↓, step length ↑, step frequency ↑, stride length ↑*					
		Kressler et al. 2014 (A)	*Post vs Mid vs Pre training: 10MWT ↓, 2MWT ↑*	*Post vs Mid vs Pre training: EE ↓ for 2/3 individuals, %VO2 peak ↓ for 2/3 individuals*	Post vs Mid vs Pre training: SCATS ↓, ISCIBPD sleep interference ↓, NRS_p ↓			
		Kressler et al. 2019 (B)		*HR: Ekso < OG for 2/2 individuals; VO2, EE: Ekso < OG for 1/2 individuals*				
		Cahill et al. 2018					Post training: semi structured interview not compared	
Subacute + chronic	n-RCT	Baunsgaard et al. 2018 (A)	Subacute: Post vs Pre training and FU vs Pre training: **10MWT ↓,** WISCI II ↑	*Subacute: Session 1, 12, 24:* ***HR: sitting < walking,*** *BP nc;* *Longitudinal evaluation: ****BRPE(6–20) ↘***		Subacute: Post vs Pre training and FU vs Pre: **TUG ↓, BBS ↑**		
			Chronic: Post vs Pre training and FU vs Pre training: 10MWT ↓, WISCI II ↑	*Chronic: Session 1, 12, 24:* ***HR sitting > standing,*** *BP nc* *Longitudinal evaluation:* ***BRPE(6–20) ↘***		Chronic: Post vs Pre training and FU vs Pre: **TUG ↓, BBS ↑**		
		Baunsgaard et al. 2018 (B)	FU vs Post vs Mid vs Pre training: ROM nc		Post vs Pre training: **MAS ↓**, Post vs Pre training and FU vs Pre training: ISCIBPDS ↓		Post vs Pre training and FU vs Pre training: **ISCIBDS (satisfaction item): chronic ↑,** subacte nc	
		Stampacchia et al. 2016			Post vs Pre single session: **MAS ↓, NRS_sp ↓, PSFS ↓, NRS_p ↓**		Post single session: PGIC not compared, ad hoc questionnaire for subjective experience not compared	
		Kozlowski et al. 2015	*Best performance: distance, 2MWT*	*METs: mid-time session > pre session; HR, BRPE(6–20): Sitting < Walking;* *HR, BRPE(6–20): Sitting < After Walking;* *BP variable*				N of sessions to achieve: walk, stand/sit with minimal assistance: median of 8 sessions; "contact guard" for walking and stand/sit: median of 15 and 18 session; donn/doff assistance: not compared
		Kolakowsky-Hayner et al. 2013	*Longitudinal evaluation:* *step length nc, distance ↗*		Longitudinal evaluation: SPS ↘	*Longitudinal evaluation: loss of balance ↘ and infrequent*		Longitudinal evaluation: don/doff time ↘, level of assistance provided by trained assistant during walking ↘
Unspecified TSI	n-RCT	Sale et al. 2018 (B)	*Pre vs Post training:* ***6MWT indoor/outdoor ↑; 10MWT ↓, cadence ↑, stride length ↑, velocity ↑, hip and ankle ROM ↑,*** *stance time ↓, double support time ↓*	*Post vs Pre training:* *BRPE 1–10: outdoor ↓, indoor ↑; VAS fatigue ↓*	Post vs Pre training: VAS_p ↓	*Post vs Pre training:* ***TUG ↓***	Post vs Pre training: **Satisfaction questionnaire (safety and comfort items) ↑**	
Exoskeleton: ReWalk								
Chronic	n-RCT	Asselin et al. 2015		*Single evaluation:* *HR and VO2:* ***Walking > Standing > Sitting;*** *After walking: BRPE (6–20) not compared*				
		Khan et al. 2019	*Longitudinal evaluation: steps without stopping ↗, distance ↗, walking speed ↗* *FU vs Post training vs FU:* *10MWT ↑, 6MWT ↓*	*PCI walking with ReWalk > PCI wheelchair propulsion*	Post vs Pre each sessions: NRS_p ↓ Weekly: McGill Pain Questionnaire Rating Pain Index nc, SCATS nc	**Post vs Pre training: limits of stability ↑, sway speed in sitting ↓** FU vs Post training: limits of stability ↓, sway speed ↑		
		Platz et al. 2016			Post vs Pre training: REPAS ↓	*N of sessions to achieve: standing balance 1 min with crutches not compared*	**Post vs Pre training: SF-12v2 (single role-physical domain) ↑** FU vs Post training: SF-12v2 ↑; Post training: Satisfaction questionnaire not compared	*N of sessions to achieve: sit to stand, stand to sit, walk 10 mt straight, walk 10 m straight and in curve, scend, turn around, and descend 12 steps, walk 500 m (outdoors): not compared*
		van Dijsseldonk et al. 2019						***Predictors of exoskeleton skill performance at*** ***Intermediate-skills-tests: lesion level, active lifestyle, age at injury, age at enrolment, BMI significantly correlate with EXO skill performance*** *Post vs Pre training: no predictors significantly related to exoskeleton skill performance*
		Chun et al. 2020						
		Yang et al. 2015	*Best Performance:* ***inverse relationship between level of assistance provided by trained assistant during walking and walking velocity for both 6MWT and 10MWT***					
		Benson et al. 2016	*Post vs Pre training: 10MWT ↓, 6MWT ↑*	*Post vs Pre each session: HR ↑, BP ↑, VAS (fatigue) ↑*	Post vs Pre each session:: VAS_p ↑, AS ↓	*Post vs Pre training: TUG ↓*	Post vs Pre training: ADAPSS ↓, ATD-PA ↓	
		Fineberg et al. 2013	*Single evaluation:* ***walking velocity and vGRF: SCI minimum assistanca < ABs minimum assistance***					
		Guanziroli et al. 2019	*Single evaluation:* ***10MWT: 2nd generation < 1st generation; 6MWT: 2nd generation > 1st generation***			*Single evaluation:* *STS: 2nd generation < 1st generation*		
		Zeilig et al. 2012	*Single evaluation Post training:* ***10MWT: low lesions < high lesions; 6MWT: low lesions > high lesions***	*Post vs Pre training: BP ↑, HR ↑, VAS (fatigue) ↑*	Post vs Pre training: VAS_p ↓	*Single evaluation Post training: TUG no difference between lesion level*	Single evaluation Post training: Satisfaction questionnaire not compared	
		Esquenazi et al. 2012	*Single evaluation Post training: 10MWT and 6MWT not compared*	*Post vs Pre each sessions: HR ↑, BP ↑, VAS fatigue nc*	Pre vs Post across sessions: VAS_p ↓, AS ↓		Single evaluation Post training: Satisfaction questionnaire not compared	
		Lonini et al. 2016	*Longitudinal evaluation:* ***trunk angle ↘;*** *Post vs Pre training: 10MWT ↓, Hip Flexion nc; Knee Flexion ↑, Swing Time ↓, Step Delay ↓*	*Longitudinal evaluation:* ***EE ↘***				
		Manns et al. 2019			Single evaluation Post training: semi structured interview spasticity ↓ for 4/11 individuals, pain ↓ for 2/11 individuals; FU: semi structured interview pain ↑ for 2/11 individuals			
		Talaty et al. 2013	*Single evaluation: trunk flexion (initial swing), trunk extension (entire gait cycle), hip extension (entire gait cycle), pelvis extension (entire gait cycle), knee flexion (swing), ankle plantarflexion (early stance): fast group > medium/slow group* *knee extension (stance) nc across groups*					
*Exoskeleton: Indego*								
Chronic	n-RCT	Evans et al. 2015	***6MWT: "Fast but safe" speed > comfortable speed***	***VO2 average and MET: "Fast but safe" speed > comfortable speed; %****VO2 peak and HR peak: "Fast but safe" speed > comfortable speed; walking economy: "Fast but safe" speed < comfortable speed*				
Subacute + chronic	n-RCT	Juszczak et al. 2018		*Post vs Pre training:* ***indoor BRPE(6–20) ↓,*** *outdoor BRPE(6–20) nc*	Post vs Pre training: **MAS ↓,** NRS_sp ↓		Post vs Pre training: SWLS ↑	*Post vs Pre training:* ***Donn time ↓,*** *doff time ↓*
Unspecified TSI	n-RCT	Tefertiller et al. 2018	*Post vs Mid training* ***indoor/outdoor 10MWT ↓,*** *6MWT ↑;* *Single evaluation: 600MWT not compared*			Post vs Mid training: TUG ↓		*Post vs Mid training:* ***Donn/Doff time ↓***
		Hartigan et al. 2015	*Single evaluation: 10MWT, 6MWT: not compared*					*Single evaluation: Donn/Doff Time, level of external assistance provided by trained assistant during walking: not compared*
*Exoskeleton: HAL*								
Subacute	n-RCT	Yatsugi et al. 2018	Post vs Pre training: **10MWT ↓, cadence ↓, angle of trunk swing ↓, GARS-M score ↑,** WISCI II ↑					
		Kubota et al. 2019	Post vs Pre training: **10MWT ↓, Step Lenght ↑, cadence ↑,** WISCI II ↑					
*Exoskeleton: Rex*								
Chronic	n-RCT	Birch et al. 2017				*Single evaluation: TUG not compared*	Single evaluation: Acceptability Questionaire: not compared	*Single evaluation: Time to transfer into device and level of assistance provided by trained assistant to perform 2 excersises with upper extremities: not compared*

Studies results are hierarchically reported according to the D&B total score. The type of comparison is specified within cells. In case of an increase of the data between evaluation time points “↑” is reported, while in case of a reduction of the data between evaluation time points “↓” is reported. In case of longitudinal evaluations during the training sessions "↗" is reported to indicate a progressive increase of the data while "↘" is reported to indicate a progressive reduction of the data. In case of comparison between groups or between different experimental conditions " > " or " < " are used. If no changes are reported “nc” is used. If the Authors of the study identified significant data variations, results are reported in bold characters. Italics cells indicate that evaluations were performed with the individuals wearing the EXO. For abbreviations see the Abbreviation List

**Table 6:** Results for the eight least addressed domains classified according to EXO and time since injury

		Study	Robot Data	Bowel functionality	Strength	Activities of Daily Living	Neurophysiology	Sensory function	Bladder functionality	Body composition and bone density
*Exoskeleton: Ekso*										
Subacute	n-RCT	Tsai et al. 2020	*Each session: up time, walk time and steps number: not compared*		Post vs Pre training: **LEMS ↑,** UEMS ↑	Post vs Pre training: **FIM ↑**				
		McIntosh et al. 2020	*Longitudinal evaluation: up time ↗, walk time ↗, steps number ↗*							
Chronic	RCT	Chang et al. 2018			Post vs Pre training: LEMS ↑					
	n-RCT	Gagnon et al. 2018 (A)	*Longitudinal evaluation: up time ↗; walk time ↗; steps number ↗*							
		Escalona et al. 2018								
		Sale et al. 2016 (A)		Post vs Pre training: satisfaction questionnaire (single bowel item) ↑						
		Alamro et al. 2018					*Single evaluation*: ***trunk muscle's activation: Ekso-OG > Lokomat***			
		Karelis et al. 2017								Post vs Pre training: **BMI ↑; Body weight ↑; leg and appendicular lean body mass ↑; Total, appendicular and leg fat mass ↓; Cross-sectional area of calf Muscle ↑;** Total, arm, trunk lean body mass ↑, Arm fat mass ↓, Trunk fat mass nc, Total bone mineral density ↓, Leg and tibia bone mineral density ↑, Cross-sectional area: Subcutaneous and intramuscular adipose tissue ↓
		Ramanujam et al. 2018 (A)					*Single evaluation:* *EMG lower limbs: SCI < ABs*			
		Gagnon et al. 2019 (B)								
		Ramanujam et al. 2018 (B)								
		Kressler et al. 2014 (A)					*Post vs Mid vs Pre training: EMG nc*			
		Kressler et al. 2019 (B)								
		Cahill et al. 2018								
Subacute + chronic	n-RCT	Baunsgaard et al. 2018 (A)	*Subacute*: *Longitudinal evaluation:* ***up time ↗, walk time ↗, steps ↗***		Subacute: Post vs Pre training and FU vs Pre training: **LEMS ↑**					
			Chronic: Longitudinal evaluation: **up time ↗, walk time ↗, steps ↗**		Chronic: Post vs Pre training and FU vs Pre training: LEMS ↑					
		Baunsgaard et al. 2018 (B)		FU vs Post vs Mid vs Pre training: ISCIBDS nc		Post vs Pre training and FU vs Pre training: **SCIM III > **			FU vs Post vs Mid vs Pre training: ISCIBDS nc	
		Stampacchia et al. 2016								
		Kozlowski et al. 2015	*Best performance: walk time, up time, steps number*							
		Kolakowsky-Hayner et al. 2013	Longitudinal evaluation: up time ↗, walk time ↗							
Unspecified TSI	n-RCT	Sale et al. 2018 (B)		Post vs Pre training: **Satisfaction questionnaire** (**single bowel item**) **↑**						
*Exoskeleton: ReWalk*										
Chronic	n-RCT	Asselin et al. 2015								
		Khan et al. 2019	*Londitudinal evaluation: total steps number ↗*		Post vs Pre training: UEMS ↑, LEMS ↑		Post vs Pre training: MEP ↓	Post vs Pre training: sensory key-points INSCSCI nc		
		Platz et al. 2016			Post vs Pre training: LEMS nc; UEMS nc	Post vs Pre training: SCIM nc		Post vs Pre training: sensory score INSCSCI nc		
		van Dijsseldonk et al. 2019								
		Chun et al. 2020		Post vs Pre training: Modified Lynch Gastrointestinal Survey: frequency of bowel evacuations nc, time spent on having a bowel movement nc, bowel accidents ↑, frequency of laxative and/or stool softener use ↑, overall satisfaction with bowel programs ↑; Bristol Stool Scale: stool consistency rated “ideal”↑; SCI-QOL ↓						
		Yang et al. 2015								
		Benson et al. 2016								
		Fineberg et al. 2013								
		Guanziroli et al. 2019								
		Zeilig et al. 2012		Single evaluation Post training: Satisfaction Questionnaire (single bowel item) nc						
		Esquenazi et al. 2012		Single evaluation Post training: Satisfaction Questionnaire (bowel regulation) ↑ for 5/11 individuals						
		Lonini et al. 2016	Longitudinal evaluation: **step frequency ↗ and steps number ↗ (positive correlation with session)**							
		Manns et al. 2019		Single evaluation Post training: semi structured interview faster and more regular bowel movements for 3/11 individuals; FU: semi structured interview faster and more regular bowel movements for 1/11 individuals						
		Talaty et al. 2013								
*Exoskeleton: Indego*										
Chronic	n-RCT	Evans et al. 2015								
Subacute + chronic	n-RCT	Juszczak et al. 2018		Post vs Pre training: Self reported perception: 80% individuals nc, 20% individuals ↑					Pre vs Post training: Self reported perception: 91% individuals nc, 9% individuals ↑	
Unspecified TSI	n-RCT	Tefertiller et al. 2018								
		Hartigan et al. 2015								
*Exoskeleton: HAL*										
Subacute	n-RCT	Yatsugi et al. 2018				Post vs Pre training: **BI ↑**				
		Kubota et al. 2019			Post vs Pre training: **LEMS↑**	Post vs Pre training: **FIM ↑**				
*Exoskeleton: Rex*										
Chronic	n-RCT	Birch et al. 2017								

Studies results are hierarchically reported according to the D&B total score. The type of comparison is specified within cells. In case of an increase of the data between evaluation time points “↑” is reported, while in case of a reduction of the data between evaluation time points “↓” is reported. In case of longitudinal evaluations during the training sessions "↗" is reported to indicate a progressive increase of the data while "↘" is reported to indicate a progressive reduction of the data. In case of comparison between groups or between different experimental conditions " > " or " < " are used. If no changes are reported “nc” is used. If the Authors of the study identified significant data variations, results are reported in bold characters. Italics cells indicate that evaluations were performed with the individuals wearing the EXO. For abbreviations see the Abbreviation List

**Fig. 5:**
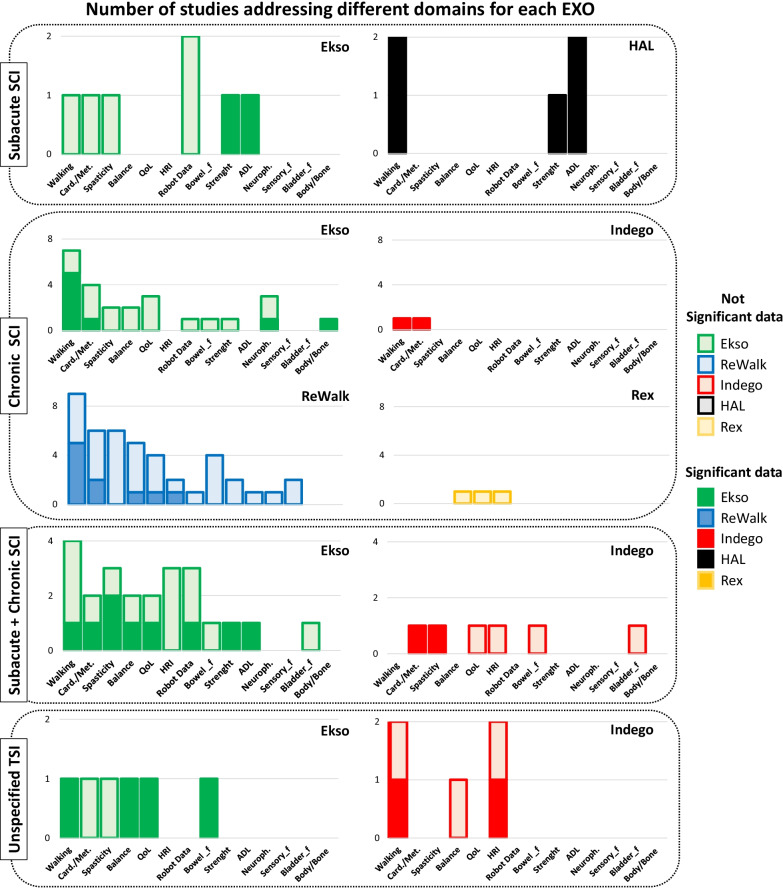
Total numbers of studies addressing the different domains for each EXO according to TSI (subacute, chronic, subacute plus chronic SCI and unspecified TSI). For each domain and for each EXO lighter tone columns represent the number of studies with no significant data reported by Authors, while darker tone columns represent the number of studies for which significant results were pointed out by Authors [*Card./Met.: Cardiorespiratory and Metabolic responses; QoL: Quality of Life; HRI: Human Robot Interaction; Bowel_f: Bowel functionality; ADL: Activities of Daily Living; Neuroph.: Neurophysiology; Sensory_f: Sensory function; Bladder_f: Bladder functionality; Body/Bone: Body composition and bone density*]

In the analysis of each domain, we verified, for each article, whether the Authors reported variations deriving from the use of EXOs and whether these variations were significant or not. Therefore, in this review we stated data as "significant" if the Authors of the included study reported significant changes in their published data. For all 14 different domains specifically addressed below, not all studies reported significant results in the different comparisons performed, as reported in Fig. 5 and Tables 5 and 6.

#### Walking domain

The pattern of outcome measures employed in the enrolled studies was extremely different, thus making comparisons unreliable. Walking velocity was measured per the Ten Meter Walking Test (10MWT) in 18 studies and per the Six Minutes Walk Test (6MWT) in 13 studies. Notably, in 11 of these studies both measures were present. Moreover, 2 studies selected the Two Minutes Walk Test (2MWT) for walking speed assessment. See Table 5.

Instrumental measures were present in 11 studies. Also, this group presented great differences, regarding both instruments and outcomes considered. All studies, except one [75], employed kinematic analyses but they varied on the measures considered. The list was extremely heterogeneous making comparison difficult (Cadence N = 7, Speed N = 5, Step length N = 5, Stance N = 4, ROM N = 4, Stride length N = 3, Double time support N = 3, Step width N = 1, Swing time N = 1).

Besides kinematics, also other instrumental measures were occasionally employed, trunk angle oscillation was assessed in 2 studies [70, 77] and vertical ground reaction forces in a single one [75]. Quite surprisingly, only 3 of the included studies used clinical scales, such as WISCI II scale alone (N = 2) or in association with the modified Gait Abnormality Rating Scale (GARS-M) (N = 1).

Different group comparisons based on 10MWT [80] as outcome measure are present in 18 studies (N = 7 Ekso [40, 42, 48–52], N = 7 ReWalk [12, 65–70], N = 2 Indego [45, 47], N = 2 HAL [77, 78]). In all these studies, regardless of EXO, training schedule or TSI, a positive trend in walking speed was observed.Three of the Ekso studies reported significant 10MWT enhancement after training. Group population included non-ambulatory individuals with chronic SCI [42] or unspecified TSI [48] ambulatory individuals with subacute SCI [52]. For ReWalk device training, different studies on chronic non-ambulatory individuals were focused on specific topics. In detail, Guanziroli et al. [68] compared two different types of ReWalk software in two groups of individuals with SCI and highlighted a better performance for the group using the second-generation software. Zeilig et al. pointed out that individuals with lower SCI walked faster than higher lesioned individuals [12] and Yang et al. demonstrated a significant inverse correlation between the level of external assistance, provided by a trained assistant, and the 10MWT data in non-ambulatory individuals with SCI [66]. Indego effects on 10MWT were addressed only in non-ambulatory individuals with unspecified TSI. Tefertiller et al. [81] pointed out a significant 10MWT improvement at the end of the training for both indoor and outdoor conditions. Hartigan et al. [47] reported data on a single session, not allowing for any comparison. The HAL device was used exclusively on subacute ambulatory participants, showing a significant 10MWT improvement at the end of the training [77, 78].

The 2MWT (N = 2 Ekso [51, 63]) and 6MWT (N = 4 Ekso [40, 48–50]; N = 6 ReWalk [12, 65–69]; N = 3 Indego [47, 76, 81]) were used for long distance speed evaluation. As for 10MWT, in the case of Ekso, ReWalk or HAL training, results showed positive effects on the walking speed measure in the 2MWT or 6MWT regardless of TSI. Studies using the Ekso device showed significant 6MWT improvement at the end of the training in participants with chronic lesion, in both non-ambulatory and ambulatory individuals [40, 50], as well as in a mixed population of ambulatory and non-ambulatory participants with unspecified TSI [48]. On the other hand, on subacute non-ambulatory individuals [49], there was an improving trend but not statistically significant. For ReWalk training, 6MWT enhancement never reached statistical significance but a positive trend was observed in Benson et al. [67]. Two studies did not allow for comparisons because the evaluation session was a single one [69] or only the best performance results were reported [66]. Comparison between individuals with low or high-level SCI lesions indicated significantly better 6MWT results in the former group [12]. Interestingly, one study indicated a significant inverse correlation between the 6MWT and the level of external assistance [66]. Regarding Indego studies a positive improvement trend of 6MWT performances was reported either in both ambulatory and non-ambulatory individuals. Furthermore, 6MWT was also employed in one study on individuals with SCI to compare comfortable vs “fast but safe” walking speed while wearing EXO, indicating significant walking improvement in the latter condition [76].

Group comparison through instrumental walking analysis varied according to the different characteristics employed. Walking speed comparison after Ekso (N = 4 Ekso [48, 50, 57, 58]) or ReWalk device usage (N = 1 ReWalk [75]) was evaluated in non-ambulatory individuals with SCI. Overall Ekso training allowed walking speed improvement, significance was present only in two studies in chronic lesion [50] or not defined TSI [48].

In two studies the walking speed parameter was also selected for comparing EXO single test in individuals with SCI vs ABs. Ramanujam et al. [57] used walking speed to compare Ekso device walking in non-ambulatory individuals with chronic lesion vs ABs. In this study ABs were required to walk with and without the Ekso device. Results showed that individuals with SCI, walk at a significantly lower speed and with a wider support surface, in comparison to ABs walking in passive modality. Fineberg et al. [75] compared ReWalk device usage in chronic non-ambulatory individuals with SCI and ABs. Individuals with SCI walked wearing EXO at a non-significant slower speed than ABs. Furthermore, the Authors classified individuals with SCI according to the level of external assistance provided by a trained assistant. Individuals with SCI able to walk with no external contact exhibited a significantly higher walking speed than individuals for which minimal physical contact was required.

The cadence parameter was analysed after training with Ekso (N = 4) [40, 48, 50, 58], ReWalk (N = 1) [70] and HAL (N = 2) [77, 78] devices, and all studies reported an improvement trend. Cadence enhancement reached significance after Ekso training in non-ambulatory individuals with chronic SCI [50] or unspecified TSI [48], as well as after HAL training in ambulatory individuals with subacute lesion [77, 78].

Stride length was assessed only in Ekso trials. The enrolled population included ambulatory and non-ambulatory individuals with chronic lesion (N = 2) [40, 58] and non-ambulatory individuals with unspecified TSI [48]. A trend of stride length improvement was observed, but it reached significance only for ambulatory individuals with chronic lesion [40] and non-ambulatory population with unspecified TSI [48].

Step length was evaluated after Ekso [40, 50, 58, 64] and HAL trainings [78]. Overall, results indicate that training allowed individuals to walk with a longer step. This improvement reached significance only for ambulatory individuals, in the case of Ekso training in chronic lesion [40] or HAL training in subacute lesions [78]. Only a single Ekso study [50] addressed step width. Non-ambulatory individuals with chronic lesion walked with a significantly larger step width after training.

Stance and double-time support phases duration alone or in combination were analysed in four Ekso studies. Pre-post training comparison was present in three studies in non-ambulatory individuals with chronic lesion [50, 58] or unspecified TSI [48]. Results were ambiguous. No significant reduction in stance and double-time support phases was reported by Ramanujan et al. [58] and Sale [48]. In another study, Sale et al. [50] reported a non-significant enhancement of the stance phase time after training. The only study reporting a significant group difference on stance time during Ekso device usage [57], reported a longer stance time duration in individuals with SCI rather than ABs. Swing phase duration was evaluated only in one study. Lonini et al. [70] reported a trend of reduction after ReWalk training.

Kinematics of the lower limb ROM was analysed in studies employing Ekso (N = 3) [48, 57, 61], ReWalk (N = 2) [43, 70] or HAL devices (N = 1) [77]. Results were extremely heterogeneous, thus making it impossible to define a common pattern (see Table 5). Significant positive effects in the reduction of trunk swing oscillation while wearing EXO were reported after ReWalk training through accelerometers in non-ambulatory individuals with SCI [70] or after HAL training by walking analysis performed without EXO in ambulatory individuals with SCI [77].The rarely used clinical scales for walking assessment were the GARS-M [82] (N = 1 HAL [77]) and the Walking Index for Spinal Cord Injury II (WISCI II) [83] (N = 2 HAL [77, 78]; N = 1 Ekso [52]). The only study with GARS-M reported a significant improvement after HAL training in subacute ambulatory individuals [77]. The studies using WISCI II reported no significant improvements after HAL [77, 78] or Ekso [52].

#### Cardiorespiratory and metabolic responses domain

Cardiorespiratory responses in individuals with SCI are of paramount importance. Nevertheless only 16 out of 41 studies included addressed this issue (see Table 5). Furthermore, data analysed and functions addressed varied across studies even if all data for each study were collected with the individuals wearing the EXO.

Heart rate (HR) was present in 10 studies (N = 5 Ekso [49, 52, 54, 59, 63], N = 4 ReWalk [12, 67, 69, 71], N = 1 Indego [76]). Data was collected in different conditions and three studies reported significant HR increase comparing sitting and standing wearing EXO (N = 2 Ekso [52, 54]; N = 1 ReWalk [71]). Two of these studies also reported a further significant HR increase comparing sitting or standing versus walking (N = 1 Ekso [52], N = 1 ReWalk [71]).

Of the 10 studies reporting HR, 6 recorded also blood pressure (BP) during Ekso training in individuals with subacute and chronic lesion or during ReWalk training in the case of chronic SCI (N = 3 Ekso [49, 52, 63], N = 3 ReWalk [12, 67, 69]). Ekso studies varied in the BP recording modality, reporting no significant changes. Conversely, studies on the ReWalk device were more uniform recording BP before and after training sessions, although no significant variations were noted.

Energy expenditure (EE) was reported in 3 studies focused on individuals with chronic SCI (N = 2 Ekso [51, 59], N = 1 ReWalk [70]). Of these studies, significant results were reported only by Lonini et al. [70]. This study reported an EE reduction after ReWalk training. The usage of different metabolic measures caused further ambiguities. Two studies employed Metabolic Equivalent Task (N = 1 Ekso [63]; N = 1 Indego [76]) and one the Physiological Cost index (N = 1 ReWalk [65]) in individuals with chronic SCI or mixed population, all reporting no significant variations after training or comparisons.

Five studies analysed oxygen consumption during EXO training, exclusively in participants with chronic SCI (N = 3 Ekso [51, 54, 59], N = 1 ReWalk [71], N = 1 Indego [76]). Significant results were obtained for Ekso [54] and ReWalk [71] devices regarding the increased oxygen consumption when transitioning from sitting to standing up to walking wearing EXO. Evans et al. [76] compared “fast but safe” vs comfortable speed oxygen consumption, during the Indego device usage, reporting a significant increase in the former condition. In addition, Escalona et al. [54] employed a wide range of parameters to analyse cardiorespiratory functions, reporting a significant increment in: carbon dioxide production, ventilation, tidal volume, respiratory rate and respiratory exchange ratio, in walking vs sitting conditions using Ekso.

Eleven studies addressed fatigue (N = 3), effort (N = 6) or both (N = 2). The five studies with Visual Analogue Scale (VAS) fatigue assessment (N = 2 Ekso [48, 50], N = 3 ReWalk [12, 67, 69]) reported variable trends after training although all of them were not significant. Eight studies analysed the perception of effort using the classical Borg Rate of Perceived Exertion (BRPE) either with the 6 to 20 or the modified version 1 to 10 scores [84] (N = 6 Ekso [48–50, 52, 63], N = 1 ReWalk [71], N = 1 Indego [46]). All studies reported a trend towards a reduced perceived effort after training. Significant BRPE reductions were reported after Ekso [52] or Indego trainings [46].

#### Spasticity domain

Spasticity and related symptoms, pain and spasms, were evaluated in 14 studies: spasticity data was present in 7, pain was reported in 12 reported pain and spasms presence in 3 (see Table 5).

Spasticity studies employed quite different outcome measures: Modified Ashworth Scale (MAS) [85] was employed in 3 studies (Ekso N = 2 [61, 62], Indego N = 1 [46]), 2 studies reported Ashworth Scale (AS) (ReWalk [67, 69]), or Numeric Rating Scale for spasticity (NRS_sp) (N = 1 Ekso [62]; N = 1 Indego [46]), and in one study the REsistance to PASsive movement Scale [86] was used (N = 1 ReWalk [72]). Finally, in one study spasticity was analyzed by a semi-structured interview (N = 1 ReWalk [44]). See Table 5. Significant spasticity reduction was observed using MAS in 3 studies, based on a mixed subacute and chronic population enrolled in Ekso [61, 62] or Indego [46] trainings. Notably, after a single training session [62] positive effects on MAS and on NRS_sp were present [62]. All remaining studies addressing ReWalk training reported a spasticity reduction trend after training [44, 67, 69, 72].

Regarding pain, different outcome measures were selected: the Visual Analogue Scale pain (VAS_p) [87], the most employed, NRS for pain evaluation (NRS_p), International SCI pain basic data set (ISCIBPD) [88], McGill Pain Questionnaire Pain Rating Index (PRI) [89], Subjective Pain Scale (SPS) and a pain semi-structured interview [44]. In all studies a positive trend in pain reduction was reported, but only in one study was significance reached.

VAS_p outcome measure is present in 6 studies (N = 3 Ekso [48–50], N = 3 ReWalk [12, 67, 69]). For both Ekso and ReWalk devices, a trend in pain reduction was observed in individuals with subacute [49] or chronic SCI [12, 50, 67, 69], as well as in a group with no TSI details [48]. Only in one ReWalk study involving individuals with chronic SCI, a trend in VAS_p increase was reported [67].

Three studies selected NRS_p (N = 2 Ekso [51, 62], N = 1 ReWalk [65]). Ekso [51] and ReWalk [65] trainings on chronic population allowed a trend in NRS_p reduction. A significant NRS_p reduction was reported after a single Ekso training session in a mixed population of both complete and incomplete lesions [62]. ISCIBPD was selected as outcome measure in 2 Ekso studies on chronic [51] or mixed population [61], suggesting a positive trend. Only one study, based on individuals with chronic lesion who underwent Rewalk training, selected PRI for pain evaluation, also reporting a positive non-significant trend [65]. One study employed the SPS as a pain outcome measure reporting a pain reduction positive trend after Ekso training in a mixed population [64]. The study of Manns et al., based on a semi-structured interview, reported no significant changes in pain after ReWalk training [44].

Spasms were seldom evaluated. Two out of three studies employed Spinal Cord Assessment Tool for Spastic Reflexes [90] in individuals with chronic SCI, indicating a positive trend after Ekso training [51] or no changes after ReWalk training [65]. A single study in individuals with both subacute and chronic SCI selected the Penn Spasms Frequency Scale [91], demonstrating a significant reduction of spasms after a single Ekso device session [62].

#### Balance domain

Twelve of the included studies addressed balance. In eight studies the Timed Up and Go (TUG) [92] was selected as outcome measure (N = 4 Ekso [40, 48, 50, 52], N = 2 ReWalk [12, 67], N = 1 Indego [81], N = 1 REX [79]). All EXOs trainings reported a positive trend in TUG performances regardless of AIS and TSI. Significant effects are reported by Sale et al. [48] in non-defined TSI non-ambulatory individuals wearing Ekso device and by Baunsgaard et al. [52] in chronic and subacute ambulatory individuals not wearing Ekso device. In the latter study, a positive effect on Berg Balance Scale (BBS) [93] was also reported.

Besides TUG, other different indexes were proposed by single studies to address balance domain. Platz et al. proposed using the number of sessions required to achieve the capability to maintain upright position wearing ReWalk device [72]. Kolakowsky-Hayner et al. [64] suggested analysing the frequency of balance loss during Ekso walking. Instrumental sitting balance assessment, limits of stability and sway speed of the Centre of Pressure (CoP), was proposed by Khan et al. [65] to evaluate ReWalk training effects in individuals with chronic lesions. Results indicated significant early improvements, which were not maintained at follow-up.

#### Quality of life (QoL) domain

Twelve studies investigated the EXO usage effects on QoL, including individuals’ perception in using EXO (N = 6 Ekso [41, 48, 50, 60–62], N = 4 ReWalk [12, 67, 69, 72] N = 1 Indego [46], N = 1 Rex [79]). See Table 5. Only five of these studies selected validated scales. International Spinal Cord Injury Basic Dataset (ISCIBDS) [61] and Patient’s Global Impression Change [62], which were administered on individuals with both subacute and chronic SCI using Ekso [61, 62] device, showed a significant improvement of self-satisfaction after training, only those participants with chronic lesions [61]. Short Form-12 v2 (SF-12 v2) [94], the Appraisals of DisAbility Primary and Secondary Scale [67] and the Assistive Technology Device Predisposition Assessment [67] were used exclusively in the case of ReWalk training for participants affected by chronic SCI. Results pointed out a positive trend of improvement in terms of health related QoL and the individual’s/EXO interaction. Interestingly, data reached significance only for the role-physical domain of SF-12 v2 [72]. Juszczak et al. [46] assessed QoL in individuals with mixed subacute and chronic SCI after Indego training, via the Satisfaction with Life Scale, showing no significant variations.

Regarding non-validated instruments, five studies used the same questionnaire consisting of 10 items about EXO training on individuals with chronic lesions (N = 1 Ekso [50]; N = 3 ReWalk [12, 69, 72]) or unspecified TSI (N = 1 Ekso [48]). Overall, a trend of positive effects in QoL was reported, highlighting emotional, physical and psychosocial benefits, as well as better comfort and stability when using EXOs. Only for Sale et al. [48] significant improvements in safety and comfort areas were obtained after Ekso training.

A non-validated semi-structured interview was used by Cahill et al. [60] at the end of the training to address Ekso device effects on QoL for individuals with chronic SCI. Individuals reported QoL benefits and a better adaptation to society in terms of physical and psychological conditions.

Participants' experience after a single session of Ekso [62] or Rex [79] devices usage, was evaluated via the non-validated questionnaires. For both studies, the acceptance of the use of the EXOs was high. The only controversial results were in relation to the simplicity of wearing the Rex device [79]. Lastly Gagnon et al. [41] proposed an online questionnaire at the end of Ekso training to participants with chronic lesion. However, no significant improvements on their perception of their health, or on their motivation to engage in physical activity were denoted.

#### Human robot interaction (HRI) domain

HRI studies have a long history over time, in terms of the roles of the robot to train, collaborate or assist humans in an intuitive and natural fashion [95]. Nevertheless, very little attention was paid to EXO usage in individuals with SCI.

The HRI was addressed by 9 out of 41 studies in terms of EXO donning/doffing time (N = 1 Ekso [64], N = 3 Indego [46, 47, 81]), the assistance provided by one or more trained assistants for donning/doffing (N = 1 Ekso [63]), for walking (N = 2 Ekso [42, 64]; N = 1 Indego [47]), and for performing upper extremity exercises (N = 1 Rex [79]), the time needed for individuals to transfer into the device (N = 1 Rex [79]), or the number of sessions necessary to reach specific motor tasks (N = 1 ReWalk [72]; N = 1 Ekso [63]). See Table 5.

Only Ekso or Indego studies addressed donning/doffing time, showing a trend in time reduction after training. This data reached significance only in two out of three Indego studies. One of these reported a significant reduction in either donning and doffing time in the case of unspecified TSI [45], while the other one only in the doffing time for a mixed population [46]. The level of external assistance provided for donning and doffing EXO was analysed by a single Ekso [63] study, as well as the time to transfer into Rex [79] device. This evaluation was performed only once, making comparisons impossible. Whit regards to the changes in the amount of assistance provided by trained assistants during walking, the usage of Ekso promoted a reduction in individuals with both subacute and chronic SCI [64] as well as in only chronic SCIs [42]. The same was observed in the Indego training in a population with unspecified TSI [47]. The number of sessions needed to reach specific motor tasks, using ReWalk [72] device (i.e. sit to stand and vice versa, walking 10 m, climbing stairs, walking 500 m outside), or to achieve the least amount of external assistance in walking, standing and sitting tasks using Ekso [63] device was analysed, with no significant information.

Lastly, Van Dijsseldonk et al. [73] studied the validity of some parameters as predictors of performances related to the use of the ReWalk device, in individuals with chronic SCI. Factors such as an active lifestyle, a young age at the time of the injury, a low lesion level and a low Body Mass Index (BMI) were found to be factors significantly correlated to the achievement of required motor tasks during training (i.e., maintenance of upright position and walking).

#### Robot data domain

The availability of recording objective performance data is one of the most claimed advantages of robotic rehab vs CPT. Nevertheless, only 8 out the 41 included studies report such data (N = 6 Ekso [42, 49, 52, 53, 63, 64]; N = 2 ReWalk [65, 70]) and no study reported data about the level of assistance provided by EXOs (see Table 6). In this selected group data is consistent when using the same EXO but varies across the different EXOs. All studies reported an improvement after training in the indexes considered, except Kozlowsky et al. [63] that reported only best performance data, making comparisons impossible. A significant improvement of up-time, walk time and steps number was reported in only one study using the Ekso [52] device, while a significant enhancement of step numbers and step frequency across sessions was pointed out ned in a single ReWalk study [70].

#### Bowel functionality domain

Bowel functionality was investigated in 8 out of 41 studies (see Table 6). Two studies used non-validated satisfaction questionnaires on individuals with chronic lesion or unspecified TSI (N = 2 Ekso [48, 50]), other two studies used satisfaction questionnaires based on the Likert Scale on a chronic population (N = 2 ReWalk [12, 69]). In the remaining 4 studies, quite different instruments were employed. In two separate ReWalk studies, not validated semi-structured interviews [44] or a battery including Modified Lynch Gastrointestinal Survey, Bristol Stool Scale and SCI-QoL Bowel Management difficulties Short Form Instrument [74] were used. The specific section of the ISCIBDS scale was also selected to assess bowel functionality after Ekso training in individuals with chronic and subacute lesions [61]. Lastly, a self-reported perception scale was employed in an Indego study [46].

Results indicate a general improvement in bowel functionality, with no significant changes regardless of TSI, EXO used or training protocols. Only one study on an unspecified TSI population using Ekso device reported a significant increase of satisfaction through a questionnaire [48].

#### Strength domain

A total of six studies evaluated strength using Lower Extremity Motor outcome measure (LEMS) [96] (N = 3 Ekso [40, 52, 53], N = 2 ReWalk [65, 72], N = 1 HAL [78]), in three of them also Upper Extremity Motor Score (UEMS) was reported [96] (N = 1 Ekso [53], N = 2 ReWalk [65, 72]). See Table 6.

All studies included reported an enhancement of muscle strength in both upper and lower limbs after EXOs training, but significant improvements were present only for LEMS, in individuals with subacute lesion in three studies either with Ekso [52, 53] or HAL [78] devices.

#### Activities of daily living (ADL) domain

Five studies evaluated ADL variations due to EXOs usage (N = 2 Ekso [53, 61], N = 2 HAL [77, 78], N = 1 ReWalk [72]). See Table 6. Function Independence Measure (FIM) scale [97], selected as outcome measures in studies using HAL [78] and Ekso [53] devices on a subacute population, showed significant improvement after training. Interestingly, a comparison between Ekso device plus CPT vs CPT alone showed lower FIM improvement when Ekso training was not provided [53].

Spinal Cord Independence Measure (SCIM) [98] was selected for ADL evaluation in two studies (N = 1 Ekso [61], N = 1 ReWalk [72]). Enhancement was observed in both cases, but significant SCIM improvements were obtained only for individuals with both subacute and chronic SCI after Ekso training [61]. Finally, Yatsugi et al. [77] used Barthel Index [99], on individuals with subacute lesion, using the HAL device, showing significant score improvements at the end of the training.

#### Neurophysiology domain

In spite of the growing interest in neurophysiological studies in the SCI field, only four of the included studies reported neurophysiological data, such as motor-evoked potentials (MEP) or electromyography, comparing different conditions or populations (see Table 6). One study assessed muscle response through MEP before and after ReWalk training, pointing out no significant changes [65]. Lower limb muscle electromyography was performed in two Ekso studies, comparing either pre vs post training [51] or ABs vs individuals with SCI [57]. Another study [55] compared muscle activation of the trunk and trunk acceleration in chronic individuals walking with Ekso device overground or on a treadmill or walking in the Lokomat. The only significant difference reported in the three studies indicated lower trunk antero-posterior and mid-lateral accelerations and lower muscle activation when using Lokomat than using Ekso devices overground.

#### Sensory function domain

Only two studies using ReWalk device on participants with chronic lesion [65, 72] investigated the possible changes in sensory circuits after training (see Table 6). Khan et al. [65] analysed the sensitivity threshold by single pulse electrical stimulation at C3-S2 sensory key points, those defined by the International Standard for Neurological Classification of SCI (ISNCSCI), with no significant modifications. Also Platz et al. [72] did not point out any significant variations on the ISNCSCI scale sensory score after training.

#### Bladder functionality domain

EXO effects on bladder functionality were investigated in two studies, on individuals with mixed subacute and chronic SCI (see Table 6). No significant effects of training were evidenced, either by using the specific section of the ISCIBDS scale (N = 1 Ekso [61]) or by participants self-reported perception data (N = 1 Indego [46]).

#### Body composition and bone density domain

Effects related to bone health and body composition were investigated exclusively in one study [56] on individuals with chronic lesion who underwent Ekso training (see Table 6). Results showed a significant increase in BMI, total body weight, leg and appendicular lean body mass and cross-sectional area of the calf muscle, as well as a reduction of total, appendicular and leg fat mass. No significant changes were evidenced in total, leg and tibia bone mineral densities (BMD).

## Discussion

This systematic review aimed to explore the effects of EXOs training on walking and SHCs in individuals with SCI to provide the current state of art on this topic.

Throughout the 41 studies included, the most addressed one was the Ekso device followed by ReWalk, Indego, HAL and Rex devices (see Fig. 4a). All studies included were of moderate or low methodological quality level (see Table 2). The low scoring was mainly due to poor study design, where control groups or follow up assessments were not included. The methodological evidence level was mostly 2B (scored by 28 of the 41 selected studies). A recent tertiary study [100], aimed to evaluate the quality of the systematic reviews based on EXOs usage in neurological disorders was carried out as a guidance for research and clinical practice. It highlighted the poor methodological and reporting quality of the studies, in spite of the recent interest in EXOs. This evidence, in line with the results of this review, emphasises the need to conduct more studies on individuals with SCI with higher methodological quality.

The analysis of epidemiological data about the SCI population showed that the studies included enrolled predominantly male subjects, with a ratio ranging from 4.76 M:1F for Indego EXO to 1.4 M:1F for HAL device (see Table 3). Despite this ratio, available published data suggest that female individuals with SCI have the same neurological and functional recovery as male ones [101]. Mean age of individuals with SCI was 43.58 ± 7.8 years: the youngest population enrolled was in the Indego studies (38 ± 3.61 years) and the oldest population was in the HAL device studies (57.25 ± 5.16 years). Even though literature suggests that age can strongly influence the onset and the evolution of SCI and related SHCs [102], the analysis of the articles showed that EXOs training effects have not been analysed taking into account different age groups. It has been demonstrated that older individuals with SCI, compared to younger ones, present a higher rate of complications, poorer neurologic recovery, and, moreover, a lower Barthel Index at discharge, level of independence in the spontaneous bladder and bowel management and frequency of independent walking [102].

Of the 541 patients enrolled, the AIS score was unknown by 4.25% and was equal to A or B by 64.33% and equal to C or D by 31.42% (see Table 3 and Fig. 1). For almost all the EXOs studies, the analysed population was mainly made up of individuals with AIS A or B, particularly for Indego and ReWalk devices. The HAL studies, instead, included only individuals with AIS C or D. Furthermore, in 44.73% of individuals with SCI the lesion level was missing. In the studies where lesion levels were reported, the thoracic SCI was the most frequent lesion (41.04%), followed by cervical (10.91%) and lumbar (3.33%) SCI. By combining data about lesion level and impairment it emerged that: in case of lesion classified as AIS A or B, EXOs training was mainly proposed to individuals with thoracic lesion (i.e. more than 50% of individuals); while in case of AIS C or D the EXOs usage was more likely proposed to individuals with cervical and thoracic lesions. For most of the studies included, the individuals enrolled within the single studies had different functional impairments, according to the AIS score, and had a wide range of lesion levels. In fact, several studies explored EXOs training effects in a population with a wide range of lesion levels, from high cervical to lumbar, or in a cohort of individuals with mixed sensory-motor, complete and incomplete SCI. For example, Zeilig et al. [12] pointed out significant differences within SCI classified as thoracic (i.e. high thoracic lesion level vs low thoracic lesion level): the lower the lesion level, the higher the walking speed on short and long distances. Taking into account previous considerations, future studies should enroll individuals with more homogeneous clinical features, in order to shed light on the relationship between EXOs training effects and impairment/lesion level. This could help in the decision-making rehabilitation process.

Data about TSI on reviewed studies were intriguing (see Table 3). The TSI of about 20% of individuals with SCI enrolled in the 41 studies was not specified. This population was mainly the one analysed in the Indego studies. The remaining population was made up of more than 50% of individuals with chronic lesions. The ReWalk and Rex studies analysed exclusively individuals with chronic SCI. On the contrary, the HAL studies enrolled only individuals with subacute SCI. Data on both chronic and subacute TSI were available only for the studies based on the Ekso device, and only Baunsgaard et al. [52, 61] directly compared data between these two groups. It emerges that there is the need to analyse the training effects in both subacute and chronic SCI for each EXO, in order to properly introduce the EXO training in the rehabilitation project of each patient.

Other heterogeneous data were related to the intervention field. The analysis of the studies included revealed a lack of homogeneity of the protocol proposed for each EXO study (see Table 3 and Fig. 3). It is reasonable to believe that EXOs training effects may depend on dosage and frequency. Unfortunately, the duration of the single treatment and the number of training sessions differed extremely among studies and sometimes not declared by Authors (see Fig. 3). For example, the number of sessions ranged from 1 to 25 for the Ekso device, from 1 to 56 for the ReWalk device, from 2 to 32 sessions for the Indego device. The two studies on the HAL device reported 5 or 10 sessions, while for the single Rex device just one session was performed. It is interesting to note that most studies on ReWalk device reported at least 24 training sessions, for the other EXOs a prevalence of the number of trainings across the studies was not identifiable (see Table 3). Moreover, by analysing the relationship between the number of sessions performed and the number of domains with significant data, it does not appear that studies with a higher number of sessions have more significant domains than those with a lower number of training sessions. For example, in the walking domain significant data were reported both in studies that carried out 1 or 2 sessions and in studies with a higher number of trainings.

A key topic in neurorehabilitation is the comparison of the effects of the EXOs usage versus CPT or other robotic-assisted gait training (e.g. robotic treadmill training). To date, the results of this review indicated that this is a field still to be investigated since no study focusing on the comparison of EXO training versus other robotic-assisted gait trainings was available, and only two studies of those included compared EXO rehabilitative effects to CPT alone [40, 53]. It is necessary indeed to underline that these comparative studies were carried out only for the Ekso device. It is interesting to point out that only 5 out of 14 domains were addressed in these two studies [40, 53] (walking, balance, strength, robot data and ADL domains), see Tables 5, 6. The single RCT included in this review, even if conducted in a very small group of ambulatory individuals with chronic SCI [40], reported a more significant improvement of step length after the Ekso training than after CPT and a significant improvement of stride length and 6MWT only for Ekso group. These walking outcomes were performed without wearing Ekso. The second n-RCT study of a larger group of individuals with subacute SCI [53], indicated a significantly higher improvement of lower limb strength and ADL in the group of Ekso plus CPT, than the CPT alone group. No significant EXO effects were noted for balance and robot data domains for both studies. All the remaining studies included did not allow comparison between treatments, mostly assessing the EXOs treatment alone (N = 37). Above data are far from conclusive. Only two Ekso studies [40, 53] compared EXO training effects versus CPT and moreover these were based on a population with different neurological features and walking abilities (see Table 3). Therefore, these studies focused on different domains, exception made for the strength one. Consequently, devoted controlled studies appear to be necessary, to deeper address all domains in a larger cohort of individuals with SCI, taking into account different neurological and performance features. In light of the foregoing, currently it is not possible to clarify whether the use of EXO devices can provide individuals with SCI with greater benefits than other types of treatment, such as CPT or other robotic-assisted gait trainings. Therefore, the potential benefits of EXO trainings should not be overestimated, despite there not being any disadvantages from EXOs usage reported in the domains analysed. The fact that EXO usage may lead to various types of adverse events and compromise the rehabilitation process, should not be overlooked.

**Fig. 6:**
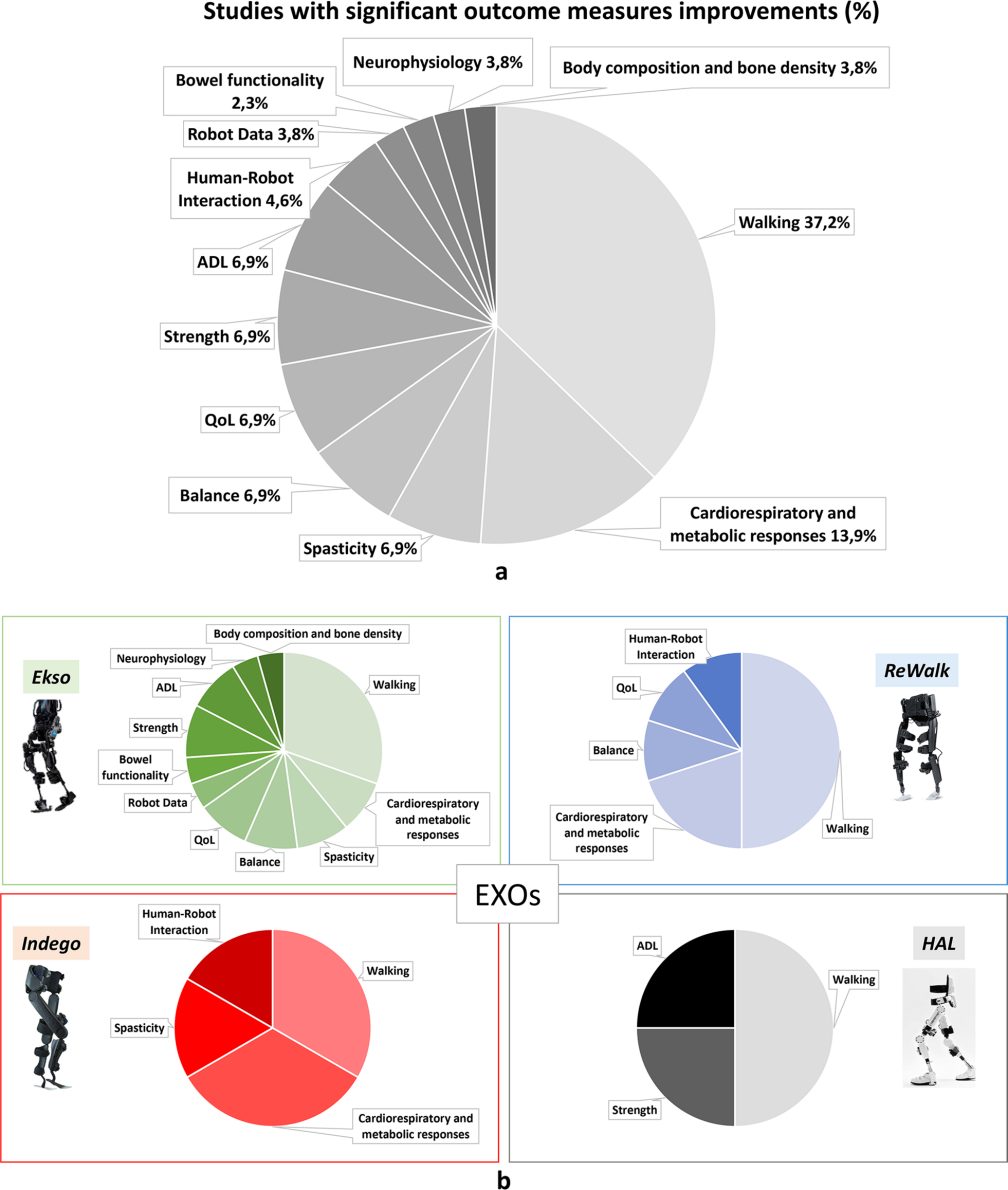
Percentage of studies including at least one outcome measures for each domain with significant improvements after EXOs training (**a**). Same data are reported in detail for Ekso, ReWalk, Indego and HAL devices (**b**)

As reported above, the outcome measures of the 41 studies were heterogeneous, covering 14 different domains, mostly related to the walking one. Only the Rex study did not show any significant training effect [79]. Figure 6 graphically reports the studies for which the Authors pointed out significant EXOs usage effects.

As for the walking domain, out of the 16 studies reporting significant effects, 10 referred to pre-post training velocity measures, 7 of which also reported significant effects on kinematic data (see Table 5). It is of relevance that the walking assessments were made either on free walking (i.e. without EXO) or in EXO assisted walking, respectively according to the ambulatory or non-ambulatory capabilities of the enrolled individuals. Overall, 6 studies reported significant effects on EXOs assisted walking velocity, and 4 on free walking velocity. These latter studies are of relevance for the expanding use of EXOs as rehabilitation devices. Ekso [40, 52] and HAL [77, 78] devices were the EXOs used in the studies reporting walking velocity improvement in ambulatory individuals. The observed population was mixed: 3 studies [52, 77, 78] on subacute and one study [40] on chronic individuals. All studies on subacute showed significant 10MWT improvement. The single study on chronic individuals with significant improvement regarded only the 6MWT [40]. Of course, the sample was too small to reach any conclusions. Nevertheless, it is interesting that short distance velocity, more related to walking abilities, improves in subacute individuals, while long-distance performance, more related to endurance, improves in chronic individuals. As for the ReWalk device, Yang et al. reported that in case of individuals who need external assistance, the lower the level of external assistance the higher the walking speed was, on short and long distances [66].

As already reported above, the ambulatory individuals were evaluated without EXO, while the non-ambulatory ones completed assessments while wearing the EXO (see Tables 5, 6). This data is related to the severity of the lesion, according to the AIS level. In fact, in case of complete SCI, individuals included in the studies were able to walk only with the EXO. Consequently, for studies involving non-ambulatory individuals, the significant data variations reported after training could be related to the training itself. The experience in using the EXO allowed individuals to be more skilled in using the device, increasing their technical skills. On the contrary, in the case of ambulatory individuals, who were able to perform assessments without EXO, the significant variations reported after training could represent a neurological improvement. In literature, variations in the AIS level or in the motor scores may be considered as indices for neurological recovery [103]. None of the studies reported AIS level variations after training, while 4 studies based on Ekso (N = 2) [40, 52] and HAL (N = 2) [77, 78] devices stated significant walking domain improvements after at least 15 Ekso device sessions and 5 HAL device sessions. Also, the strength domain significantly improved after training in two out of these four studies (N = 1 Ekso [52], N = 1 HAL [78]). Furthermore, for the study [52] where walking and strength domains improvements were reported, Ekso training benefits were also maintained at the follow up examination, reinforcing the hypothesis of a possible motor recovery owing to EXO training. Considering that the above reported studies involved individuals with subacute lesion, future studies with a control group that also include neurophysiological evaluations are necessary to estimate if the neurological recovery is in fact due to EXO training. Indeed, EXO training is based on a bottom-up approach that acts on the lower limbs (bottom) through the acquisition of technical skills and aimes at influencing the neurological system (top) by exploiting residual neural plasticity mechanisms. On the other hand, also a top-down approach should be considered, in fact an increasing number of studies support the hypothesis that technological devices allow a more direct action on the central nervous system to recover peripheral functions [104]. To define the relationship between neurological recovery and EXO training, the possibility that the type of EXO, the treatment protocol and the level of assistance provided by the EXO could influence training effects on motor recovery, should be considered.

Besides the EXOs effects on gait, Ekso or ReWalk trainings also influence dynamic balance (see Table 5). The Ekso device training allowed balance benefits both in chronic and subacute ambulatory participants [52], who therefore carried out the assessments (i.e. BBS and TUG) without wearing the EXO, and in those who are not ambulatory, with unspecified TSI [48], who carried out the assessment (i.e. TUG) while wearing Ekso. Also, trunk balance may be influenced positively by EXO training. The ReWalk gait training increased trunk control in the sitting position in non-ambulatory chronic individuals [65]. This evidence suggests that, although training with EXOs has walking as its primary objective, EXOs usage may also allow for enhancements in balance and trunk muscle training. This would bring about consequent effects on autonomy and ADL management, but it needs to be confirmed by devoted studies.

For most commercial EXOs, some walking aids (crutch/es, cane/s, rollator) are required to improve balance for a safe management of the device [2], suggesting possible effects on upper limb strength alongside the benefits on the lower limb ones. Although muscle strength is almost always used to evaluate the effects of walking training in trials based on the SCI population, it is interesting that very few studies selected UEMS or LEMS for the evaluation of the strength domain. The results on the strength domain pointed out no EXO training effect on UEMS. LEMS significantly improved after training in three studies (N = 2 Ekso [52, 53]; N = 1 HAL [78]) in subacute ambulatory individuals, while in chronic SCIs, neither ambulatory nor non-ambulatory, no EXO training improvement was noted (see Table 6). Given these results, we can assume that in the subacute phase it is possible to utilize EXOs to increase strength in the lower limbs. This would be reinforced by the possibility that the LEMS’ increase is reported for individuals who underwent training with EXOs, for which it is possible to adjust the level of assistance provided by EXOs. Since the studies included in this review did not analyse the effects on strength of the assistance provided by EXOs, it could be interesting for future trials to explore the relationship between it and the potential strength improvements. In fact, although EXOs allowed for the possibility to adjust the level of assistance provided, it is curious that no study reports data about the variation of assistance across training sessions. Having this type of information would be useful to better tailor ad-hoc EXOs training. We can presume that for individuals with incomplete SCI, assistance can be gradually reduced as functional recovery increases. Only the Ekso studies provided assistance information, but not in all the articles included. As reported in the training section, training progression was personalized for each individual according to walking modalities (i.e. First Step, ProStep, ProStep +), the level of EXO assistance or the variations in walking aids. With regards to the level of EXO assistance, it is stated that the Ekso device was initially set at the maximum assistance level, according to the individual’s capabilities, to encourage the individuals’ contribution to the movement. Furthermore, it is stated that an initially high level of assistance was progressively lowered, according to the individual’s increased performance.

In addition to the level of assistance provided by EXOs, other performance data during walking were collected from the studies reviewed. In particular, robot data was available only for Ekso and ReWalk devices (see Table 6). During EXOs training, using either Ekso or ReWalk devices, a progressive increase of up-time, walk time and steps number was reported, which allowed participants to gradually manage the device better. Nevertheless, significant improvements of these parameters were observed only in a single study on the Ekso device [52] across training sessions. These performance data may reflect the ability of EXO users to properly manage the device and may influence the HRI. Few studies included analysed the HRI domain, mainly reporting the time spent for donning and doffing the device, suggesting that individuals with SCI who underwent Indego training became significantly faster in wearing and removing it [46, 47, 81] (see Table 5). Although HRI studies have a long history over time, future studies aimed to specifically evaluate the interaction between individuals with SCI and EXOs are needed.

Considering that EXOs training induces movements of lower limbs by providing sensory inputs, these rhythmic movements could induce the reorganization of the spinal and supraspinal circuitry, as well as a possible decrease of spasticity in SCI [105], a common symptom after SCI [11]. No study on Rex and HAL devices was available in the spasticity domain, but results from studies based on Ekso, ReWalk and Indego EXOs suggest a general trend of positive training effects on spasticity (see Table 5). In a few studies, a significant reduction of spasticity in lower limb muscles was observed in individuals with complete and incomplete, subacute and chronic SCI, after Ekso [61] and ReWalk [46] training. Interestingly, Stampacchia et al. [62] demonstrated that even a single session using the Ekso device allowed individuals with mixed TSI to significantly reduce MAS, pain and spasms. These two symptoms are closely linked to spasticity and are related to the individual’s perception of physical and emotional functionality after SCI, as well as chronic fatigue and decreased QoL [106]. Considering that pain can persist for years after SCI, a major impediment to effective rehabilitation, the positive effects of powered EXOs on pain could be of particular interest. However, before starting any trial, it would be useful to classify in depth the type of pain to assess. In fact, different types of pain such as neuropathic or visceral pain, as well as pain linked to over exercising, incorrect posture, poor biomechanics or sores, could co-exist after SCI.

Another common SHC due to SCI is bone loss and the resulting osteoporosis [107]. Bone loss predisposes individuals with SCI to fractures [108–110]. Bone loss is caused by a combination of factors including changes in bone metabolism, blood circulation abnormalities and reduction in mechanical forces from both weight-bearing activities and muscle contractions [111]. Subsequently, it has been hypothesized that weight-bearing in EXO may improve the progressive loss of BMD [56]. Nevertheless, only a single study on chronic complete lesioned individuals using the Ekso device addressed this issue [56] and reported no significant changes in bone health (see Table 6). Besides this data, it is interesting to note that one Ekso study and one ReWalk study reported bone fracture as an adverse event, mainly for individuals with complete SCI. A meta-analysis reported the incidence of bone fracturing at any time during EXO training program [28]. Furthermore, Van Herpen et al., identified the misalignment of the EXO joints, relative to the user joints, as one of the main causes for lower limbs fracture, especially in osteopenic or osteoporotic bones [112]. Understanding the relationship between fracture risk and specific levels of BMD for each EXO device would help clinicians to select individuals with SCI suitable to train with EXO. Currently it is difficult to define a BMD threshold for exclusion from EXO usage and, despite the fracture predisposition of the individuals with SCI, not all the EXO studies included the BMD as an inclusion/exclusion criteria. Such as data suggests that an appropriate screening of bone condition should be performed before EXO training, considering osteoporosis or osteopenia as a relative contraindication for EXO usage.

However, results indicate that Ekso training has positive effects on BMI, on lean vs fat mass and on total body weight. These latter results are interesting considering that obesity is a major risk factor of cardiovascular disease and that is frequently found in SCI population, due to the decreased physical activity/exercise, the decrease in lean body mass, and the increase in fat mass [108, 113]. Furthermore, the types of cardiovascular training are limited for individuals with SCI, due to their paralysis and the necessary effort in traditional non-robotic walking orthoses. This is why EXOs may provide a viable alternative.

A significant increase of HR and oxygen consumption was reported during a single session of Ekso [52, 54] and ReWalk [71] training, in individuals with chronic and complete SCI, but only during the transition from sitting or standing to walking while wearing EXO. This increase can be considered as the normal response to maintaining the BP when changing position [114] that would take place even without wearing an EXO. In fact, no significant BP variations were reported. In spite of this, it is uncertain if the EXO training increases or even decreases the BP response in individuals with SCI as compared to overground walking, because no control group was included in any of the Ekso and ReWalk device studies mentioned. Nevertheless, we can speculate that, apart from the physiological cardiovascular system adaptation, the active contribution of upper limbs and trunk necessary for weight shifting and dynamic balance control during EXOs walking may also influence HR and oxygen consumption. Devoted studies to address if EXOs usage can serve as an effective means of cardiovascular exercise need to be done. It is interesting to note that even if fatigue was explored by almost all studies included in the cardiorespiratory and metabolic parameters domain, only Baunsgaard et al. [52] and Juszczak et al. [46] highlighted a reduction in the effort perceived after training, respectively using Ekso and Indego devices (see Table 5). This suggests a better management of these EXOs by the user.

Other SHCs are those related to the pelvic floor, such as bladder and neurogenic bowel dysfunctions, with consequent constipation and/or incontinence [7]. In this review, few studies covered bowel and bladder domains. No EXO training effects on bladder functionality were evidenced, while some changes on bowel functionality were reported (see Table 6). However only Sale et al. [48] obtained a significant improvement in perceived bowel functionality after Ekso training, in unspecified TSI population. These results are in line with a previous study, that recommended EXOs training to improve, in particular, bowel functionality/management and related QoL measures [11], using both upright posture and overground walking exercise. It should be pointed out that only one single study selected bowel functionality and management as the primary goal [74], which took into account the frequency of bowel evacuations, time spent on bowel management, bowel accidents and laxative dosage. On the contrary, the other studies addressed the bowel domain per the single items of not specific questionnaires or semi-structured interviews. Therefore, specific scales and/or questionnaires for bowel assessment and management in the SCI field are still needed. This is even more important because bowel dysfunction was second, in order of importance, only to loss of mobility [74] in the list of the domains that individuals with SCI addressed as having impact on their QoL. Curiously, none of the studies focused on the SCI sexual component, when SCI causes neurogenic sexual dysfunction, and their QoL is mainly affected by sexual function [115], as well as the bowel and bladder ones [116].

Most individuals with SCI witness a decrease in their QoL because of the difficulties performing self-reliant ADL and in taking part in everything a community may offer. Health-related QoL has been investigated more than the ADL domain and it was addressed for all EXOs, except for the HAL device, including either subacute or chronic SCI (see Table 6). Data has demonstrated that the Ekso device is perceived to be safe and comfortable, with a consequent high level of user satisfaction [48], for chronic SCIs only. One single study considered QoL “*satisfaction with life*” after Ekso usage [61]. It improved in individuals with chronic SCI; on the contrary, no changes were reported in subacute cases of SCI. One explanation could be that QoL is known to improve over the years following the injury, suggesting a process of adaptation over a long period [9]. One study investigated the physical role domain (“*how much one thinks she or he can accomplish and how much one feels to be limited in the kind of work or other activities*”) in using ReWalk device [72]. It is well known that individuals with SCI are judged for their diminished physical functioning whit respect to the general population, but their self-perception of possible achievements, rather than limitations, during their activities was positively changed after the EXO training. It is reasonable to think that standing, being mobile in an upright posture and experiencing the possibility to “overcome” a simple wheelchair-based mobility, gave the trained individuals with SCI a different perspective on what they can physically achieve. Taking into account that these aspects were rarely addressed, we suggest evaluating motivational, psychosocial, and emotional aspects when examining individuals with SCI that want to use an EXO to walk.

Even if the main goal of EXOs training is not directly to improve ADL, EXOs trainings may help individuals with SCI to achieve independence in ADL and reduce secondary co-morbidities [53] because of the possibility to increase movement performances and independence in self-care. In this review, few studies measured the EXOs effects on ADL, pointing out improvements: on the BI [77] after HAL device usage, on the SCIM III after Ekso device training [61] and on the FIM [78] after both HAL and Ekso device usage [53] (see Table 6). This last article is the only one that selected the individual functional activity performance scale as the primary outcome measure. It is interesting to see that these few studies focused mainly on a subacute population. The ADL improvements, greater in subacute SCI, could probably be attributed to early phase improvements following SCI. Higher quality studies, with appropriate control interventions, need to be conducted to deeper address the relationship between ADLs improvements and EXOs usage in a subacute population. In fact, the absence of a control group could obscure the potential benefit of EXOs intervention. Achieving maximal independence in ADL in people with SCI is strongly related to their health and well-being, and it has been shown that participation in social activities leads to a higher QoL [117]. Despite this relationship between ADL and QoL, only one study examined both domains, also considering the TSI (see Table 6). While the ADL progress appeared greater after EXOs training in the subacute phase, QoL improvements were predominant in chronic SCI cases. This suggests two paths for future studies on the EXOs training: one is to better analyse the relationship between QoL and ADL, and the other one is to classify the results of these domains according to TSI.

Lastly, none of the studies included in this review addressed the impact of the EXOs on rehabilitation costs, despite the growing interest in this field. However Pinto et al. [118], in a single study, that has not met the inclusion criteria of this review, analysed how the EXOs usage affected hospital budgets. It suggested that it reduced hospital costs. If this observation is confirmed by future targeted studies, clinicians will be motivated to implement the EXOs usage in the clinical daily routine. However, it is not yet clear if the EXOs usage is more effective than CPT, taking into account the lack of studies which compare EXOs versus CPT. In this context, even if Pinto et al. suggested that the EXOs usage may be linked to reduction in hospital costs, the comparison between EXO trainings and CPT should be deeper analysed to better understand the cost-effectiveness.

### Limits

This systematic review is indirectly limited by the poor or moderate methodological quality of the studies included, by the small heterogeneous number of participants with variable dosage of interventions, by the presence and/or absence of control groups and/or follow-up assessments in only few studies and by the various parameters adopted in each domain for different types of comparisons. Consequently, trying to find significant data could be inconclusive. In light of the above, we suggest future devoted studies based on statistical analysis that combines data from multiple studies, with the goal to address and deeper clarify the same scientific question. We do so in consideration of our approach of reporting the significant data, with the absence of significant disadvantages due to EXO training, also in order to avoid an overestimation of the benefits of the EXO usage.

## Conclusion

In light of the results of this systematic review, it appears that the strengths and weaknesses of EXOs are starting to be defined in scientific literature, even if a clear evidence about the full range of possible EXOs benefits or detriments have not been established yet. Results of this systematic review suggested that the EXO training could allow potential benefits in different domains, even if adverse events (e.g. skin lesion, bone fracture at lower limbs, …) may occur. However, these benefits need to be confirmed through specific high-quality RCTs. In fact, the small number of studies with a control group addressed few domains and did not allow to establish whether the benefits deriving from the use of EXO are greater or lesser than CPT. Furthermore, studies targeting those domains less addressed, need to be carried out. Also, studies focusing on homogenous epidemiological and clinical features up to date have been either partially carried out, or not at all. Lastly, from the studies included it seems there is no direct relationship between dosage and domains’ improvements. To actually clarify this point, further studies are needed to compare the effects of different EXO dosages according to the EXO device, the TSI and the severity of the lesion. Further in depth studies of the above mentioned key points could help the clinicians to better select the appropriate training for individuals with SCI.

## Supplementary Information

**Additional file 1.** Exclusion Criteria of individuals with SCI. Studies data are hierarchically reported according to Downs and Black tool score.

## Data Availability

Not applicable.

## Abbreviations

10MWT: 10 Meter Walk Test
2MWT: 2 Minutes Walk Test
6MWT: 6 Minutes Walk Test
600MWT: 600 Meter Walk Test
ABs: Able Bodied subjects
ADAPSS: Appraisals of DisAbility Primary and Secondary Scale
ADL: Activities of Daily Living
AIS: Asia Impairment Scale
AS: Ashworth Scale
ATD-PA: Assistive Technology Device Predisposition Assessment
BBS: Berg Balance Scale
BMD: Bone Mineral Density
BMI: Body Mass Index
BP: Blood Pressure
BRPE (1–10): Original Borg Rate of Perceived Exertion
BRPE (6–20): Modified Borg Rate of Perceived Exertion
C: Cervical
CoP: Centre of Pressure
CPT: Conventional Physical Therapy
D&B: Downs and Black tool
EE: Energy Expenditure
Ekso-OG: Ekso-Overground
EMG: Electromyography
EXO: Exoskeleton
F: Female
FIM: Functional Independence Measure
GARS-M: Gait Abnormality Rating Scale-modified
HAL: Hybrid Assistive Limb
hr: Hour
HR: Heart Rate
HRI: Human Robot Interaction
ISCIBDS: International Spinal Cord Injury Basic Dataset
ISCIBPD: International Spinal Cord Injury Basic Pain Dataset
ISNCSCI: International Standard for Neurological Classification of SCI
L: Lumbar
LEMS: Lower Extremity Motor Score
LOI: Level of Injury
M: Male
MAS: Modified Ashworth Scale
MEP: Motor Evoked Potentials
n-RCT: Non Randomized Controlled Trial
NRS_p: Numeric Rating Scale Pain
NRS_sp: Numeric Rating Scale Spasticity
PCI: Physiological Cost Index
PGIC: Patient’s Global Impression Change
PRI: Pain Rating Index
PRSIMA: Preferred Reporting Items for Systematic Reviews and Meta-Analyses
PSFS: Penn Spasms Frequency Scale
QoL: Quality of Life
RCT: Randomized Controlled Trial
ROM: Range of Motion
RER: Respiratory Exchange Ratio
RR: Respiratory Rate
SCATS: Spinal Cord Assessment Tool for Spastic Reflexes
SCI: Spinal Cord Injury
SCIM: Spinal Cord Independence Measure
SCI-QoL: Spinal Cord Injury-Quality of Life bowel management difficulties short form instrument
SF-12v2: Short Form Health Survey
SHC: Secondary Health Condition
SPS: Subjective Pain Scale
STS-Time: Sit-to-stand Time
SWLS: Satisfaction Whit Life
T: Thoracic
TSI: Time Since Injury
TUG: Timed Up and Go
UEMS: Upper Extremity Motor Score
VAS: Visual Analogue Scale
VAS_p: Visual Analogue Scale Pain
VAS_sp: Visual Analogue Scale Spasticity
VCO2: Carbon Dioxide Production
VE: Ventilation
VO2: Oxygen Consumption
VT: Tidal Volume
WISCI II: Walking Index for Spinal Cord Injury II
wk: Week
yr: Year
