# Supplementary material for: Overground robotic training effects on walking and secondary health conditions in individuals with spinal cord injury: systematic review
Source: J Neuroeng Rehabil. 2022 Mar 15;19:27. doi: 10.1186/s12984-022-01003-9 (PMC8922901; doi:10.1186/s12984-022-01003-9)
Supplement: Supplementary file 1 — Additional file 1. Exclusion Criteria of individuals with SCI. Studies data are hierarchically reported according to Downs and Black tool score. [file 12984_2022_1003_MOESM1_ESM.pdf]

# SUPPLEMENTARY MATERIALS: Exclusion Criteria

| Exclusion Criteria | | | |
|---|---|---|---|
| | | **Study (D&B total score)** | **Exoskeleton: Ekso** |
| SUBACUTE | n-RCT | **Tsai et al, 2020 (15)** | Uncontrolled cardiovascular conditions, orthostatic hypotension, progressive SCI, upper leg length discrepancy> 1.27cm or lower leg discrepancy>1.9 cm, ulcerations, MAS=4, cognitive impairments, pregnancy, colostomy, mechanical ventilation, other medical issues |
| | | **McIntosh et al, 2020 (10)** | Untreated or active lower extremity fractures, medical instability |
| CHRONIC | RCT | **Chang et al, 2018 (18)** | Lower motor neuron injury, severe neurologic injuries other than SCI, severe comorbiditie, pressure ulcers, or any skin issues, severe osteoporosis; MAS≥ 3 in the lower extremities, uncontrolled clonus, unstable spine, unhealed limb or pelvic fractures, ROM restrictions, upper extremity strength deficits, heterotopic ossifications, contractures, psychiatric or cognitive comorbidities, any physical therapy intervention within 3 months prior to the study |
| | n-RCT | **Gagnon et al, 2018 (A) (14)** | Previously training with robotic overground EXOS, other nervous system damage, impaired skin integrity, concomitant or secondary musculoskeletal impairments, lower extremity fracture, unstable cardiovascular or autonomic system, cognitive or communication problems |
| | | **Escalona et al, 2018 (11)** | Other neurological disorders, injuries to the skin in areas of contact with the EXO, psychiatric or cognitive impairments, poorly controlled spasticity of the lower extremities |
| | | **Sale et al, 2016 (A) (11)** | Heart or respiratory comorbidity, hemodynamic instability, unhealed fractures, heterotopic ossification, osteoporosis, heeight below 157.48 cm or above 187.96 cm, weight above 100 Kg, cognitive and/or communicative disability |
| | | **Alamro et al, 2018 (10)** | NR |
| | | **Karelis et al, 2017 (10)** | Other nervous system damage aside from the SCI, impaired skin integrity, concomitant or secondary musculoskeletal impairment, cardiovascular disease, cognitive dysfunction |
| | | **Ramanujam et al, 2018 (A) (10)** | Joint contractures limiting ROM during walking with assistive devices, medical issue preventing full weight bearing and walking, skin issues, problems wearing an external device that supported the spinal column or the head, neck, or trunk, yypotension, pressure sores, pre-existing condition affecting ability to exercise, hospitalization for heart problems within 3 months before the study, bone density measurement below 0.5755 gm/cm2 |
| | | **Gagnon et al, 2018 (B) (9)** | Previously overground EXO training, other nervous system damage, Impaired skin integrity, other musculoskeletal impairments, lower extremity fracture within the past year, unstable cardiovascular or autonomic system, cognitive or oral communication problems |
| | | **Ramanujam et al, 2018 (B) (9)** | NR |
| | | **Kressler et al, 2014 (A) (8)** | Any surgery within the preceding 3 months, recent lower extremity fracture, participation in lower limb exercise conditioning or pressure ulcer within past 3 months, upper limb pain that limited weight bearing on forearm crutches, pregnancy, diabetes, MAS>3, unresolved deep vein thrombosis, uncontrolled autonomic dysreflexia |
| | | **Kressler et al, 2019 (B) (7)** | ROM restrictions, upper extremity strength deficits, deep vein thrombosis, decreased standing tolerance, significant osteoporosis, uncontrolled spasticity, uncontrolled autonomic dysreflexia, skin integrity issues, cognitive impairments, pregnancy, colostomy, impaired balance |
| | | **Cahill et al, 2018 (2)** | Inability to provide informed consent, who hadn't used the exoskeleton in a gym-based setting, who were ambulatory |
| HRONIC | | **Baunsgaard et al, 2018 (A) (13)** | Previous training with exoskleton, Spinal instability, Acute deep vein thrombosis, Autonomic dysreflexia, Heterotopic ossification, Hip subluxation, Cognitive deficits, Neurological injury, Pregnancy |
| | | **Baunsgaard et al, 2018 (B) (13)** | Previous training with EXO, spinal instability, acute deep vein thrombosis, autonomic dysreflexia, heterotopic ossification, hip subluxation, cognitive deficits, neurological injury, pregnancy |

| | | | |
|---|---|---|---|
| SUBACUTE + CI | n-RCT | **Stampacchia et al, 2016 (11)** | Asymmetry in limb length or any important limitation of the ROM in hip, knee or ankle, psychiatric disorders |
| | | **Kozlowski et al, 2015 (10)** | Weightbearing restrictions, spinal instability, unresolved deep vein thrombosis, osteoporosis that prevents safe standing or may increase the risk of fracture caused by standing or walking, uncontrolled autonomic dysreflexia, skin integrity issues on surfaces that would contact the device or on buttocks, limited ROM |
| | | **Kolakowsky-Hayner et al, 2013 (7)** | Known spinal instability, history of long bone fracture below the neurological level of injury, lower extremity joint instability, other neurological disorder, psychiatric or cognitive impairment, skin lesions, pregnancy, poorly controlled spasticity, orthostasis, autonomic dysreflexia |
| UNSPECIFIED TSI | n-RCT | **Sale et al, 2018 (B) (10)** | Heart or respiratory comorbidity, hemodynamic instability, unhealed fractures, heterotopic ossification that may impede walking, osteoporosis, height below 158 cm or above 188 cm, weight above 100 Kg, cognitive and/or communicative disability |
| | | | **Exoskeleton: ReWalk** |
| CHRONIC | n-RCT | **Asselin et al, 2015 (14)** | Neurological impairment other than SCI, Recent lower-limb fracture or proximal femur BMD t-score at the distal femur <3.5, BMD at the proximal tibia <2 g/cm2, pressure ulcers of the trunk and lower limb, contractures at the hip and knee greater than 20°, heterotopic ossification in the joints of the lower limbs, pregnancy |
| | | **Khan et al, 2019 (14)** | Comorbidities that interfere with training or measurements such as severe head injury, Bone fractures within the last 2 years, Low bone density, Contractures, Active pressure sores, Severe spasticity |
| | | **Platz et al, 2016 (14)** | Diagnosis of neurological injury other than SCI, Severe concurrent medical disease, illness, or condition, lower extremity fracture within the past 6 months, trunk and/or lower extremity pressure ulcerss, severe spasticity, significant contractures, heterotrophic ossification of the lower extremities, psychopathology, other illnesses, pregnancy |
| | | **van Dijsseldonk et al, 2019 (13)** | MAS>3, restricted ROM in the hip, knee, or ankle joint, unable to control crutches, to make a transfer from a chair to a wheelchair without the use of external support, osteoporosis, fractures of the lower extremities in the last 2 years, pregnant or lactating, balance disorders, neurogenic heterotopic ossification, history of other conditions that could interfere with the motor learning process |
| | | **Chun et al, 2020 (12)** | Paralyzing neurological condition other than SCI, severe medical illness, disease or condition, lower extremity fracture in the past 2 years, Hip T-score <3.5 or BMD <0.60 g/cm2 at the distal femur and/or proximal tibia, MAS=4, lower extremity heterotopic ossification, trunk, pelvic or lower extremity pressure injury, flexion contracture limited to 35° at the hip and/or 20° at the knee, psychopathology that may conflict with study objectives, untreated/uncontrolled hypertension (systolic blood pressure >140 mmHg, diastolic >90 mmHg), Pregnancy and/or lactation |
| | | **Yang et al, 2015 (12)** | Diagnosis of neurological injury other than SCI, severe concurrent medical disease, illness, or condition, lower extremity fracture within the past 2 years, DXA <-3.0 at the lumbar spine and bilateral proximal femurs, Knee BMD <0.70 g/cm2, systemic or peripheral infection, stherosclerosis, congestive heart failure, or history of myocardial infarction, pressure ulcers, other illness, MAS >4, significant contractures, heterotropic ossification of the lower extremities |
| | | **Benson et al, 2016 (11)** | History of severe neurological injuries other than SCI, concurrent medical, unstable spine or unhealed limbs or pelvic fractures, ROM <90° at hip and knee joints, severe spasticity or uncontrolled clonus, diagnosis of severe osteoporosis/-penia, psychiatric or cognitive conditions that may interfere with the trial, pevious use of any EXO robotic device, inabuility to provide informed consent. |
| | | **Fineberg et al, 2013 (11)** | NR |
| | | **Guanziroli et al, 2019 (11)** | History of severe neurological disorders, concurrent severe medical disease, pressure sores, unstable spine, unhealed limb or pelvic fractures, psychiatric or cognitive status that may interfere with the trial |
| | | **Zeilig et al, 2012 (11)** | History of severe neurological disorders, concurrent severe medical disease, pressure sores, unstable spine, unhealed limb or pelvic fractures, psychiatric or cognitive status that may interfere with the trial |
| | | **Esquenazi et al, 2012 (10)** | Severe neurologic injuries/medical diseases/osteoporosis/spasticity, unstable spine, heterotopic ossification, cognitive problems, contractures |

| | | | |
|---|---|---|---|
| | | **Lonini et al, 2016 (9)** | NR |
| | | **Manns et al, 2019 (6)** | Comorbidities that interfere with training or measurements, fractures within the last 2 years, BMD t-score <= 3, hip, knee and ankle contractures >10°, active pressure ulcers, severe spasticity |
| | | **Talaty et al, 2013 (3)** | Osteoporosis, hemodynamic instability, ROM of hip, knee and ankle non adequate, MAS > 3, skin problems |
| | | | **Exoskeleton: Indego** |
| **CHRONIC** | **n-RCT** | **Evans et al, 2015 (9)** | Skin comprise, pregnancy, poor bone health, lower extremity MAS>3, unresolved deep vein thrombosis or autonomic dysreflexia, contractures |
| **SUBACUTE + CHRONIC** | **n-RCT** | **Juszczak et al, 2018 (10)** | NR |
| **UNSPECIFIED TSI** | **n-RCT** | **Tefertiller et al, 2018 (12)** | Heterotopic ossification, moderate to severe traumatic brain injury, inability to follow instructions, pregnancy, lower extremity joint limitations > 10° at hips, knees, or ankles |
| | | **Hartigan et al, 2015 (9)** | NR |
| | | | **Exoskeleton: HAL** |
| **SUBACUTE** | **n-RCT** | **Yatsugi et al, 2018 (12)** | NR |
| | | **Kubota et al, 2019 (11)** | Inadequately controlled cardiovascular and respiratory disorders, intellectual impairments, moderate-to-severe joint disorders, moderate-to- severe involuntary movements, impairments of postural reflexes in the trunk or lower limbs |
| | | | **Exoskeleton: Rex** |
| **CHRONIC** | **n-RCT** | **Birch et al, 2017 (9)** | NR |

Studies data are hierarchically reported according to the Downs and Black Tool score.
